# PEG-induced physiological drought for screening winter wheat genotypes sensitivity – integrated biochemical and chlorophyll *a* fluorescence analysis

**DOI:** 10.3389/fpls.2022.987702

**Published:** 2022-10-12

**Authors:** Vesna Peršić, Anita Ament, Jasenka Antunović Dunić, Georg Drezner, Vera Cesar

**Affiliations:** ^1^ Department of Biology, Josip Juraj Strossmayer University of Osijek, Osijek, Croatia; ^2^ Department of Small Cereal Crops, Agricultural Institute Osijek, Osijek, Croatia; ^3^ Faculty of Dental Medicine and Health, Josip Juraj Strossmayer University of Osijek, Osijek, Croatia

**Keywords:** triticum aestivum, PEG-6000, photosynthesis, free proline, lipid peroxidation, HAC (hierarchical agglomerative clustering), PCA

## Abstract

This study aimed to screen different winter wheat genotypes at the onset of metabolic changes induced by water deficit to comprehend possible adaptive features of photosynthetic apparatus function and structure to physiological drought. The drought treatment was the most influential variable affecting plant growth and relative water content, and genotype variability determined with what intensity varieties of winter wheat seedlings responded to water deficit. PEG-induced drought, as expected, changed phenomenological energy fluxes and the efficiency with which an electron is transferred to final PSI acceptors. Based on the effect size, fluorescence parameters were grouped to represent photochemical parameters, that is, the donor and acceptor side of PSII (PC1); the thermal phase of the photosynthetic process, or the electron flow around PSI, and the chain of electrons between PSII and PSI (PC2); and phenomenological energy fluxes per cross-section (PC3). Furthermore, four distinct clusters of genotypes were discerned based on their response to imposed physiological drought, and integrated analysis enabled an explanation of their reactions’ specificity. The most reliable JIP-test parameters for detecting and comparing the drought impact among tested genotypes were the variable fluorescence at K, L, I step, and PI_TOT_. To conclude, developing and improving screening methods for identifying and evaluating functional relationships of relevant characteristics that are useful for acclimation, acclimatization, and adaptation to different types of drought stress can contribute to the progress in breeding research of winter wheat drought-tolerant lines.

## 1 Introduction

Plants’ susceptibility to water deficit is genetically predetermined in molecular, biochemical, physiological, and phenological properties, while plant water status regulates the intensity of physiological processes (Boyer, 1996; Tuberosa, 2012; Lawlor, 2013; Ribeiro Reis et al., 2020). Partitioning of assimilates and reproductive success of plants is influenced mainly by water use and plant water status, making it the primary driver of yield under drought stress (Blum, 2009). Therefore, water deficit induces numerous biochemical and physiological responses affecting plant growth by modifying its anatomy and morphology (Reddy et al., 2004; Shao et al., 2008). These development limitations mainly happen due to photosynthesis-dependent reductions in carbon balance (Flexas et al., 2009). Therefore, crop cultivars improved to withstand water deficit possess distinct physiological adaptive traits directed mainly to support yield under drought. Although drought usually occurs at different intensities and crop growth stages, it is relatively less common during seedling development. Seedling survival becomes vital in seasonal rainfall lag and can be linked to yield performance under drought (Agbicodo et al., 2009; Blum, 2011b). The most apparent basis for seedling survival is an osmotic adjustment, allowing hydration retention in low water potential to sustain photosynthesis *via* turgor maintenance (Blum, 2005; Blum, 2011a; Blum, 2017).

Nevertheless, drought score at the seedling stage is considered an irrelevant indicator of grain yield because recovery and damage repair in young plants can still enable future gain in grain yield (Blum, 2005; Blum and Tuberosa, 2018); thus, the relevance of seedling research becomes less significant. However, the importance of seedling survival for genetic engineering is an opportune trait. After all, seedling survival is easier to assess and demonstrate since seedlings are not subjected to the complexities of development and reproduction, unlike fully developed plants (Blum, 2011a). Moreover, selection based on seedlings research gains importance regarding environmental conditions vital for seedlings establishment, like germination in the limited water supply. Today’s changes in the frequency and occurrence of extreme weather conditions are also causing additional disturbances in plants’ water absorption, despite sufficient soil water. Thus, physiological drought can be caused by high or low soil temperatures, increased salinization, reduced air humidity, and increased airflow intensity (Novák, 2009), emphasizing the importance of seedling research.

Photosynthesis is one of the plants’ most essential and sensitive processes, which any minor stressful event can disrupt. Its efficiency is critical in determining genotypes’ resistance to any stress. Inhibition of photosynthesis in water deficit conditions correlated well with reduced water potential and stomatal conductance (Flexas and Medrano, 2002; Flexas et al., 2004; Chaves et al., 2008; Flexas et al., 2016) and decreased level of relative water content (Lawlor, 2002). Mild to moderate drought stress causes the stomata to close, promoting a reduction in net photosynthesis to avoid additional water loss. However, closed stomata reduce ribulose-1,5-bisphosphate carboxylase/oxygenase supply with CO_2_, favoring its oxygenase function (Chaves and Oliveira, 2004), thus correlating with the loss of ATP (Lawlor and Tezara, 2009). The inability to utilize light energy then creates an imbalance in the electron transport chain (Foyer et al., 2012), increases the production of reactive oxygen species (Miller et al., 2010), affects the ratio of photosynthetic pigments (Li and Kim, 2022), and leads to the disorganization of thylakoid membranes (Zhu et al., 2021), which are the first to respond to even the slightest disturbance in the functioning of the plant (Stirbet and Govindjee, 2011). It is well known that drought impacts the plant’s photosynthetic apparatus (Goltsev et al., 2012; Jedmowski et al., 2013; Jedmowski et al., 2015; Kalaji et al., 2018; Bashir et al., 2021). Accordingly, drought causes changes in the redox state of PSI, impairs an electron transfer at both the acceptor and the donor side of PSII, affects the oxygen-evolving complex, and decreases energetic connectivity and electron transfer capacity (Zhou et al., 2019).

Compared to PSI, PSII has good resistance to drought, and permanent adverse effects on PSII are present only in extreme drought conditions (Lauriano et al., 2006; Desotgiu et al., 2012). Besides, photosynthesis has shown resilience and high stability of the quantum yield of primary photochemistry of PSII when exposed to various intensities of drought stress (Oukarroum et al., 2007; Oukarroum et al., 2009; Qi et al., 2021). Widely applied measurements of chlorophyll *a* fluorescence help detect these first non-visible changes in photosynthetic apparatus functioning and structure (Strasser et al., 2004b; Goltsev et al., 2009; Goltsev et al., 2016; Kalaji et al., 2016; Kalaji et al., 2018; Samborska et al., 2019). Apart from being a simple, *in vivo*, and susceptible method, the fluorescence measurement provides a large amount of information on the physiological state of plants, which is essential for investigating and explaining physiological changes in certain environmental conditions like nutrient deficiency (Živčák et al., 2014; Samborska et al., 2019; El-Mejjaouy et al., 2022; Lotfi et al., 2022), salt (Kalaji et al., 2011b; Dąbrowski et al., 2016, Dąbrowski et al., 2017; Khatri and Rathore, 2022), temperature (Yang et al., 2009; Kalaji et al., 2011a; Oukarroum et al., 2016; Mihaljević et al., 2020) or drought stress (Zivcak et al., 2008a; Oukarroum et al., 2009; Goltsev et al., 2012; Goltsev et al., 2016; Kalaji et al., 2016; Kalaji et al., 2018). Many papers show that the measurement of chlorophyll fluorescence can potentially be used as a method of screening sensitive and tolerant genotypes of a particular plant species (Oukarroum et al., 2007; Boureima et al., 2012; Guha et al., 2013; Jedmowski and Brüggemann, 2015; Banks, 2018; Chiango et al., 2021; Markulj Kulundžić et al., 2022).

The complex information obtained by fast chlorophyll fluorescence kinetics can be presented in several ways. A typical fluorescence transient shows phases from the onset of illumination (F_0(50μs)_) to a maximal possible fraction of closed RCs (F_M(P)_) value, which is defined as the OJIP curve, and analyzed by JIP-test (for detailed literature review, cf. (Strasser and Strasser, 1995; Stirbet and Govindjee, 2011; Goltsev et al., 2016; Tsimilli-Michael, 2020). For various intensities of drought impact, among obtained parameters, photosynthetic efficiency indices (PIs) have proven to be very useful for screening plants and evaluating the overall effect of stress on photosynthetic performance, while individual expressions provided pieces of information on the impact on separate processes (Tsimilli-Michael and Strasser, 2013; Živčák et al., 2014; Kalaji et al., 2017; Tsimilli-Michael, 2020). Furthermore, double normalized differential chlorophyll *a* fluorescence data, especially in the form of L- (ΔW_OK_) and K-bands (ΔW_OJ_), were used to assess the plant’s resistance to drought-induced stress (Oukarroum et al., 2007; Oukarroum et al., 2009; Brestic et al., 2012; Brestic and Zivcak, 2013; Guha et al., 2013; Kalaji et al., 2018; Zhou et al., 2019).

When developing drought-resistant genotypes, it is essential to understand the physiological processes concerning photosynthesis and transpiration when water is limited. Precisely because of the complex genetic control of drought tolerance, it is necessary to test the performance of all varieties at different stages and intensities of drought. A plant’s response to a lack of water depends on the duration and severity of the water deficit and the time of occurrence. Numerous studies have shown the connection between seed germination, seedling establishment, and soil moisture (Bouaziz and Hicks, 1990; Farsiani and Ghobadi, 2009; Jabbari et al., 2013; Lamichhane et al., 2018). Unlike fully grown plants, seedlings are not subjected to long-term environmental influences. They can use all the potentials of plant primordia to turn distressed conditions into beneficial stress indicative of adaptation (Kranner et al., 2010). Although germination and the first stage of the seedling establishment are among the most vulnerable plant growth stages, they are also prerequisites for the success of crops since the physiological traits of early seedling growth can be transferred to later stages of their life cycle. Some studies have shown that drought during the first stages of growth can efficiently diminish drought stress in the following stages of plant development (Selote et al., 2004; Abid et al., 2018; Auler et al., 2021). Selecting cultivars based on their drought tolerance in the first stages of development, where the problem is water scarcity in the early season, can help improve crop yields (Ahmed et al., 2020; Lu et al., 2022; Ru et al., 2022). Thus, making the development of drought tolerant crops environmentally and economically important.

This research aimed to establish a reliable screening of 18 winter wheat genotypes for drought susceptibility by comparing the impact of PEG-induced physiological drought on morphological, biochemical, and physiological characteristics of seedlings shoots and roots. Furthermore, the aim was to identify possible photosynthetic mechanisms which best explain the variability among genotypes and could serve to differentiate and describe the seedlings’ response to imposed physiological drought conditions. Therefore, this study can further upgrade our understanding of water-stress physiology, contributing to the progress in breeding research of winter wheat drought-tolerant lines.

## 2 Materials and methods

### 2.1 Plant material and experimental conditions

Eighteen genotypes of winter wheat (*Triticum aestivum* L.) were obtained from Agricultural Institute Osijek, Croatia (L459-2012, Osk 54/15, Osk 78/14, Osk 108/04, Osk 251/02, Osk 70/14, Osk 52/13, Osk 106/03, Osk 114/08, Osk 120/06, Osk 84/15, Osk 102/03, Osk 51/15, Osk 111/08, Osk 4.40/7-82, Osk 44/11, Osk 381/06, L259-2009) to study the effect of drought at the seedling stage. All genotypes have good tolerance to low temperatures, lodging, and winter wheat diseases. A widely used polymer polyethylene glycol 6000 (PEG-6000, ACROS Organics™) was used to simulate the impact of drought stress. PEG is chemically inert and non-toxic for plant cells and changes the water potential of solutions by inducing potential osmotic pressure. For each treatment and replicate, 50 healthy seeds were hand sorted, soaked in water for 5 h, and surface sterilized with 2.5% sodium hypochlorite to prevent mycosis. Washed seeds were inoculated aseptically on moist filter papers (GE Healthcare Whatman™ Grade 598) in Petri dishes and placed in the dark for 72h at 20°C for germination. Germinated seeds with emerged radicles and coleoptile were transferred on a half-strength Hoagland’s nutrient solution (Hoagland and Arnon, 1950). Water potential (ψ, MPa) was adjusted with PEG-6000 for control (ψ = -0.033 MPa) and drought-induced stress (ψ = -0.301 MPa) conditions according to Michel and Kaufmann (Michel and Kaufmann, 1973). All experimental units were placed in a controlled climate chamber under a 16/8h light/dark photoperiod at 22°C, 70/75% relative humidity, and light intensity of 120 μmol m^-2^ s^-1^ (CWL and TLD 36W, Philips) for 7 days enabling slow development of stress as the most desirable since it simulates natural conditions. A constant temperature was used for the growth conditions since PEG water potential can variate with temperature. The growth medium was replaced daily throughout the experiment. Wheat seedlings of different genotypes were grown in a completely randomized design with three replicates of each treatment, and the experiment was replicated twice. All subsequent measurements were made on the first fully developed leaf and roots of 10-day-old seedlings.

### 2.2 Initial screening for drought tolerance - PEG test

A slightly modified PEG test was used for initial drought sensitivity screening (Agarie et al., 1995; ElBasyoni et al., 2017). Ten small leaf cuttings, approximately 1 cm in length, of 10-day-old wheat seedlings were placed in 50 ml test tubes and washed with deionized water three times. The leaf cuttings were then submerged in 20 ml of PEG-6000 solution (ψ = -0.602 MPa) for dehydration treatment (P) or deionized water as the control (C). The test tubes with samples were then placed in the dark for 24h at room temperature, and conductivity (µS cm^-1^) was measured afterward using the Conductivity Meter (Mettler Toledo). Next, the leaf cuttings were washed rapidly three times with deionized water. Both the control and treatment samples were submerged in 20 ml of deionized water and placed in the dark for another 24h at room temperature for rehydration. After the rehydration, conductivity was measured, and leaf tissue was killed by heating the samples for 20 min at 100°C. The final conductivity was measured after cooling to room temperature. Three replicates were analyzed for both the control and PEG treatment. Cell membrane stability of wheat seedlings was expressed as cell membrane integrity percentage (%) with higher rates indicating less damage using the equation: $CMI\;\left(\%\right)=\left[\left(1-\frac{P_{int}}{P_{tot}}\right)/\left(1-\frac{C_{int}}{C_{tot}}\right)\right]\;\times 100$ , where and are the sum of conductivity measurements of the PEG desiccation treatment and the control after dehydration and rehydration, and and are the final conductivity measurements after the tissue destruction by heating.

### 2.3 Determination of morphological, physiological, and biochemical indices

#### 2.3.1 Growth measurements

Seedlings were harvested on the 10^th^ day to determine the growth parameters. The straight ruler method was used to determine the height of seedlings. Each seedling’s longest primary seminal root was measured (Image J). The dry weight of roots and shoots was measured after drying in an oven for 24 h at 80°C.

#### 2.3.2 Relative water content

The relative water content of leaves (RWC) was determined in random leaves that were cut into approximately 1 cm long pieces, weighted fresh (FW, g), and placed to float on distilled water until fully rehydrated (approx. 4h) in the dark, weighted to obtain turgid weight (TW, g) and then dried until a constant oven-dry weight (DW, g) is obtained (at 80°C for 24 h). The equation described by Turner et al. (Turner, 1986) was used to calculate the percentage of relative water content: *RWC* (%) = (*FW* − *DW*)/(*TW* − *DW*) × 100.

#### 2.3.3 Electrolyte leakage

Electrolyte leakage (EL) was determined in random leaves cut to leaf segments (approx. 1 cm length) by placing them in closed vials containing 20 ml of deionized water for 24h at room temperature in the dark. Relative EL of the samples was estimated according to the ratio of the initial conductivity (EC_1_, µS cm^-1^) to the absolute conductivity after heat disruption of cell membranes (100°C, 20 min, EC_2_, µS cm^-1^) with the equation: *EL* (%) = (*EC*_1_/*EC*_2_)× 100 .

#### 2.3.4 Malondialdehyde and free proline content

For all genotypes and treatments, the lipid peroxidation and free proline content were determined in the leaves and roots of wheat seedlings. Lipid peroxidation was estimated by measuring the amount of malondialdehyde (MDA) produced by the thiobarbituric acid (TBA) reaction (Heath and Packer, 1968). Approximately 0.2 g of homogenized fresh tissue sample was extracted in 0.1% trichloroacetic acid (TCA). The mixture of extract and 0.5% thiobarbituric acid in 20% TCA was heated at 95°C for 30 min, then quickly cooled in an ice bath, and the absorbance was recorded at 532 (specific) and 600 (non-specific) nm (UV-VIS Spectrophotometer, Analytic Jena SPECORD 40). After subtracting the non-specific absorbance, the MDA content was calculated using its molar extinction coefficient (ϵ_532_ = 155 mM^-1^ cm^-1^), and the results were expressed as nmol (MDA) g^-1^ dry weight.

Free proline was analyzed by the ninhydrin-based colorimetric assay (Abrahám et al., 2010). Plant material (approximately 0.1 g of a homogenized fresh tissue sample) was extracted with 3% sulfosalicylic acid. The reaction mixture of proline extract, 3% sulfosalicylic acid, glacial acetic acid, and acidic ninhydrin was incubated at 95°C for 60 min. The reaction was terminated on ice. The red-colored chromophore was extracted with toluene, and the absorbance of the toluene fraction was measured at 520 nm. The free proline amount expressed as μmol g^-1^ of dry weight was calculated using a standard curve for L-proline.

#### 2.3.5 Chlorophyll pigments

Carotenoids (Car), chlorophyll *a* (Chl *a*), and chlorophyll *b* (Chl *b*) of wheat seedlings were determined according to (Lichtenthaler and Buschmann, 2001). Pigments from fresh leaf samples (0.1 g) were extracted with pure acetone with several re-extractions, centrifuged each time at 18 000 × g and 4°C for 15 min. The absorbances of the extracts were recorded at 470, 644.8, and 661.6 nm and calculated using the following equations:


$$Chl\;a\;\left(mg/ml\right)\;=\;11.24\;\times A{\;}_{661.6}–\;2.04\times A_{644.8}$$

$$Chl\;b\;\left(mg/ml\right)\;=\;20.13\;\times A_{644.8}\;–\;4.19\;\times A_{661.6}$$

$$Car\;\left(mg/ml\right)\;=\;\left(1000\;\times A_{470}\;–\;1.90\times \;Chl\;a\;–\;63.14\times \;Chl\;b\right)/214$$

### 2.4 Measurement of the chlorophyll *a* fluorescence transient (O-J-I-P)

The emission of the chlorophyll *a* fluorescence was measured on the first fully developed leaf of randomly chosen 20 plants for every genotype and treatment. The measurements were performed in leaves previously adapted to the dark for 30 min with a Handy PEA fluorometer (Hansatech, UK). The transient was induced with a red-light pulse of 3000 μmol m^-2^ s^-1^ and analyzed using the JIP-test (Strasser and Strasser, 1995; Stirbet and Govindjee, 2011; Goltsev et al., 2016; Tsimilli-Michael, 2020). For a detailed evaluation of the OK, OJ, JI, and IP phases, a transient curve was normalized as a relative variable fluorescence at time t, as follows: , where is the fluorescence yield (Stirbet et al., 2014). The kinetic differences were calculated from the relative variable fluorescence by subtracting the transient of stressed and control plants. For detailed definitions and explanations of the JIP test parameters, see (Goltsev et al., 2016) and (Tsimilli-Michael, 2020).

### 2.5 Statistical analysis

The Shapiro-Wilks test was used to check if the data followed normality, and Levene’s test was used to check the assumption of equal variances. Since the assumptions were not rejected, two-way ANOVA and Tukey HSD tests were used to determine significant genotype differences. To better observe the differences between the treatment and the control group and individual genotypes, the difference between the treatment’s mean value and the control group’s mean value was calculated. The calculation of the mean difference does not consider the standard deviation within the groups. Therefore, a quantitative measure of the strength of an effect (Hedges effect size) was calculated as the standardized mean difference between two groups ( $\bar{x_{1}}-\bar{x_{2}}$ ) based on the pooled, weighted standard deviation ( of the sampled population ( ) according to Hedges and Olkin (1985):


$$d=\left(\bar{x_{1}}-\bar{x_{2}}\right)/SD_{pooled}$$

$$SD_{pooled}=\sqrt{\left(\left(n_{1}-1\right)SD_{1}^{2}+\left(n_{2}-1\right)SD_{2}^{2})/(n_{1}+n_{2}-2\right)}$$

Considering that this paper deals with data obtained in a laboratory experiment and small independent samples, an unbiased version of effect size was derived according to Ellis (2010):


$$corrected\;\left(Hedges\;d\right)\;≅d\left[1-(3/\left(4\left(n_{1}+n_{2}\right)-9\right)\right]$$

Effect size assesses the degree to which the examined effect is present or the degree to which the null hypothesis is not valid, so it is not just binary data. In other words, if the null hypothesis is correct, the P-value indicates the probability that the observed difference exists. But also, P-values can indicate how incompatible the data are with a statistical model. A statistically insignificant result does not “prove” the null hypothesis. Neither statistically significant results “prove” any other hypothesis. Suppose we supplement the P-values obtained by testing the null hypothesis with the P-value from the test of a predetermined alternative (such as the minimum important effect size). In that case, we will get a better and more informative representation of the proven values (Nakagawa and Cuthill, 2007). The higher the effect size, the greater the increase of a parameter in the treatment compared to the control group. Negative effect size values indicate a decrease in a parameter compared to the control group. The large effect depends on the context and known sources of variability (Sawilowsky, 2009; Sawilowsky et al., 2011). All calculations using previously described equations: pooled SD, biased effect size, 95% confidence intervals, and statistical analyses from which these results were derived (p-value for the mean difference using 2-tailed T-test) were done in Excel (Microsoft Corporation, 2019). Effect size estimates with 95% confidence intervals were graphically presented by stock graphs (high-low-close) in combination with line plots of the mean difference.

Principal Component Analysis (PCA), a multivariate statistical technique, was used to reduce a large set of chlorophyll *a* fluorescence parameters to the most informative ones (Goltsev et al., 2012; Kalaji et al., 2017). PCA was also used to investigate the effect of genotype diversity on the structure of the variability in measured fluorescence parameters and their correlations with morphological and biochemical parameters with direct oblimin rotation. To classify the variability in response to mild drought stress among genotypes into groups, a hierarchical k-means clustering algorithm on main features was used to obtain optimal cluster solutions (Bussotti et al., 2020). PCA and HAC multivariate statistical analysis and graphical presentations of PCA and HAC were made with XLSTAT 2022.2.1.1304 (Addinsoft, 2022).

## 3 Results

### 3.1 Initial screening for drought tolerance – PEG test

For a preliminary screening of winter wheat varieties to drought susceptibility, seedlings of 18 wheat genotypes were subjected to a PEG test as an efficient method to determine drought sensitivity. Cell membrane stability as the integrity percentage is shown in **Table 1**. Although desiccation treatment significantly increased electrolyte leakage in all genotypes (cell membrane integrity ranged from 41 to 69%) and differences (One-way ANOVA, F_17,90_ = 2.4, p = 0.005) among genotypes were found, the Tukey HSD test revealed that significant difference exists only between genotype with the lowest (Osk 106/03) and the highest cell membrane integrity (Osk 4.40/7-82, 114/08, 51/15, 108/04 and 381/06). Based on these results and to find phenotypic variability among genotypes, the potential osmotic pressure of PEG-6000 for the experimental treatment was reduced from moderate to mild drought stress (to -0.301 MPa).

**Table 1:** The initial screening and ranking of 18 different winter wheat genotypes based on cell membrane stability of wheat seedlings expressed as cell membrane integrity (CMI %) obtained by PEG test.

	Genotype	CMI %	SE	CI (95%)		Tukey HSD
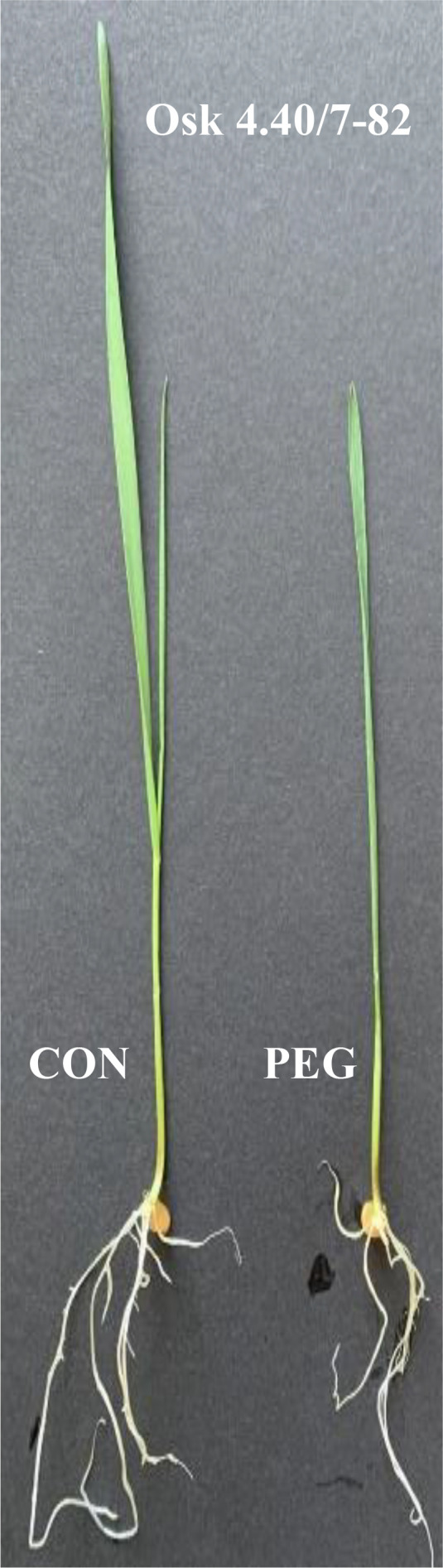
	Osk 106/03	43.8	1.4	41	46.7	a
	Osk 52/13	49.7	2.2	45.4	54	ab
	Osk 78/14	51.6	0.5	50.6	52.7	ab
	Osk 102/03	52.6	0.9	50.9	54.3	ab
	Osk 70/14	52.9	0.9	51.1	54.6	ab
	L459-2012	53.4	2.4	48.6	58.2	ab
	Osk 44/11	54.4	1	52.4	56.5	ab
	Osk 120/06	55.4	2.5	50.5	60.3	ab
	Osk 251/02	55.6	2.8	50	61.3	ab
	L259-2009	56.1	2.7	50.7	61.4	ab
	Osk 84/15	56.4	2.3	51.9	61	ab
	Osk 111/08	56.5	2	52.5	60.5	ab
	Osk 54/15	56.9	1.4	54	59.8	ab
	Osk 4.40/7-82	57.1	1.3	54.5	59.6	b
	Osk 114/08	57.5	4	49.6	65.4	b
	Osk 51/15	59.5	2.8	53.9	65	b
	Osk 108/04	59.7	2.4	55	64.5	b
	Osk 381/06	60.2	4.5	51.3	69.2	b

The results are the mean, standard error (SE), and 95 % confidence interval (CI). Means followed by a joint letter are not significantly different (the Tukey HSD-test at the 5% significance level). On the left side is an example of 10-days-old wheat seedlings (Osk 4.40/7-82) exposed to test conditions: control (CON, ψ = -0.033 MPa) and physiological drought (PEG, ψ = -0.301 MPa). Different hues of blue, yellow and red color scale were used for visualisation of CMI % data (min, average, max).

### 3.2 Morphology and relative water content

**Figure 1:**
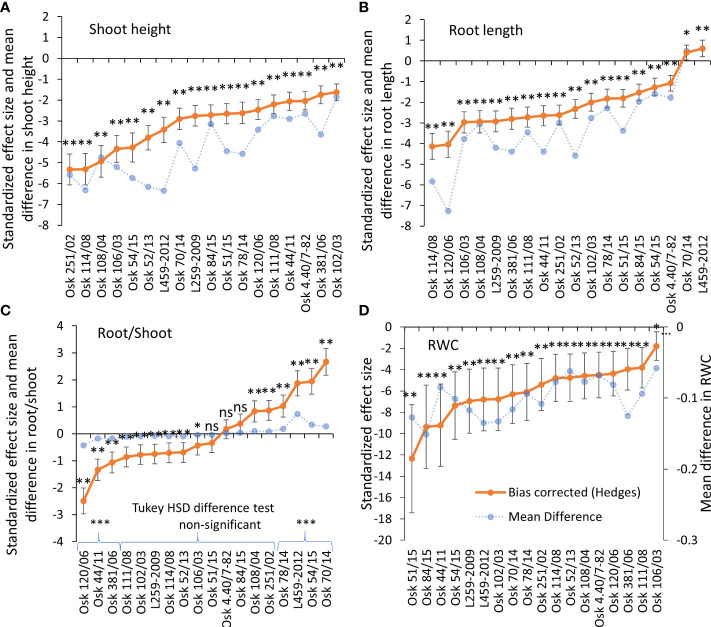
Hedges bias-corrected treatment effect size (with confidence interval) and the mean difference in shoot height **(A)**, root length **(B)**, root-to-shoot ratio **(C)**, and relative water content (RWC) **(D)** between PEG-induced drought and control treatment in 18 genotypes of wheat seedlings. Significant effects of PEG-induced drought are marked with asterisks (* p<0.05, ** p<0.01, *** p<0.001), and ns stands for non-significant (p > 0.05). Based on the Tukey HSD difference test, significant differences were determined in root-to-shoot ratio for Osk 120/06, 44/11, and 381/06 (decreased root/shoot induced by drought) and for Osk 78/14, 54/15, 70/14, and L459-2012 (increased root/shoot ratio induced by drought).

Examining the influence of genotypic variability and drought treatment on plant growth, two-way ANOVA showed a significant effect of the tested factors: genotypes (p<0.001), PEG induced drought (-0.03 and -0.3 MPa; p<0.001) and their interactions (p<0.001) on the shoot and root growth, as well as their ratio, with treatment as the most influential variable that affects plant growth, and the interaction with genotype variability as the most significant variable that affected the root-to-shoot ratio (**Table 2**). Shoot height was significantly reduced by PEG-induced drought in all genotypes (**Figure 1A**, Tukey HSD, p< 0.05), with a decrease ranging from 16% (Osk 102/03) to 53% (L459-2012) compared to the control plants. A statistically significant negative effect of drought on the root growth was observed in most of the tested genotypes except for L459-2012 and Osk 70/14 (**Figure 1B**), which in contrast to all the others, showed an increase in root length (by 11% and 9%).

**Table 2:** Two-way ANOVA analysis of the effects of wheat cultivars and drought treatment on plant growth.

	df	Shoot height	Root length	Root/shoot ratio
**F** **R^2^** **Genotypes (G)** **Treatment (T)** **G×T**	35, 2101 17 1 17	223.72*** 0.78 138.82*** 4839.45*** 27.44***	150.63*** 0.72 94.03*** 2531.22*** 53.18***	55.41*** 0.48 38.89*** 12.89*** 72.99***

*** (P< 0.001).

Significant differences (F values) are marked with asterisks, and df are degrees of freedom.

The differences in shoot and root growth were reflected in their ratio. The Tukey difference test determined a non-significant difference between control and drought in the root-to-shoot ratio of eleven cultivars. However, the calculated standardized effect size (Hedges d) revealed only three cultivars with a non-significant change in the ratio (**Figure 1C**). The most substantial increase in the root-to-shoot ratio under drought was found in the genotype L459-2012 (by 161%), although not the highest effect due to more considerable variation among the measured plants. At the same time, the most substantial decrease was found in Osk 120/06 (by 45%). Three groups of wheat response in the root-to-shoot ratio can be discerned, the ones with decreased ratio (120/06, 44/11, 381/06), the ones with very little to no change in the ratio (eleven cultivars, **Figure 1C**), and the ones with significantly increased root-to-shoot ratio (70/14, 54/15, L459-2012, 78/14). In all varieties, at least a double increase in root dry matter was observed in conditions of PEG-induced drought (**Supplementary Material Table 2**). In addition to the increased accumulation of carbohydrates (since 50% of dry weight refers to carbohydrate content), an increase in osmolytes or secondary compounds like phenols and lignin is also possible (Ghanbari and Sayyari, 2018; Qayyum et al., 2021). Relative water content was also decreased (on average by 10%) in all genotypes when exposed to physiological drought, with no significant differences among genotypes referring to treatment as the most influential variable (**Figure 1D**, **Supplementary Material Table 3**).

### 3.3 Free proline and lipid peroxidation

**Figure 2:**
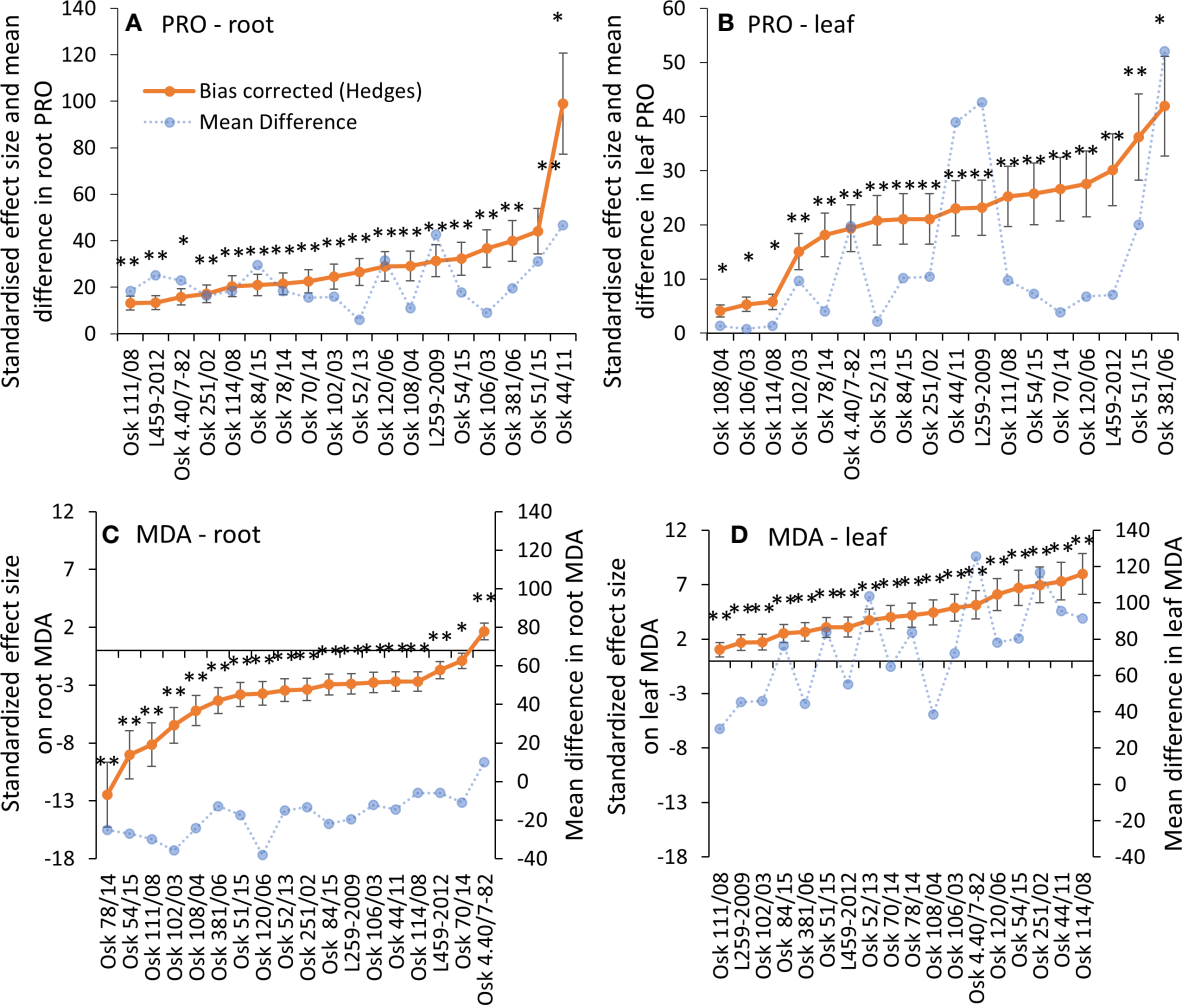
Hedges bias-corrected treatment effect size (with confidence interval) and the mean difference between PEG-induced drought and control treatments for proline and MDA contents in roots **(A, C)** and leaves **(B, D)** of 18 genotypes of winter wheat seedlings. Significant effects of PEG induced drought are marked with asterisks (* p< 0.05, 0.01, ** p< 0.001), and ns stands for non-significant (p > 0.05).

A significant increase in PRO was induced by physiological drought in both roots and leaves of all genotypes (**Figures 2A, B** **)**. The most considerable mean differences and the effect sizes in leaves were found for Osk 381/06 (**Figure 2B**). The two-way ANOVA model explained more than 99% of the data with a significant treatment effect, genotype variability, and interaction in leaves and roots. Based on the Type III sum of squares, the most influential was genotype variability (**Supplementary Material Table 3**). As for the malondialdehyde content, MDA decreased in roots (except Osk 4.40/7-82) in almost all genotypes when exposed to PEG-induced physiological drought. At the same time, there was a significant increase in leaf MDA content (**Figures 2C, D** **)**. Also, the two-way ANOVA showed that genotype variability is the most influential variable (**Supplementary Material Table 3**).

### 3.4 Pigment content

**Figure 3:**
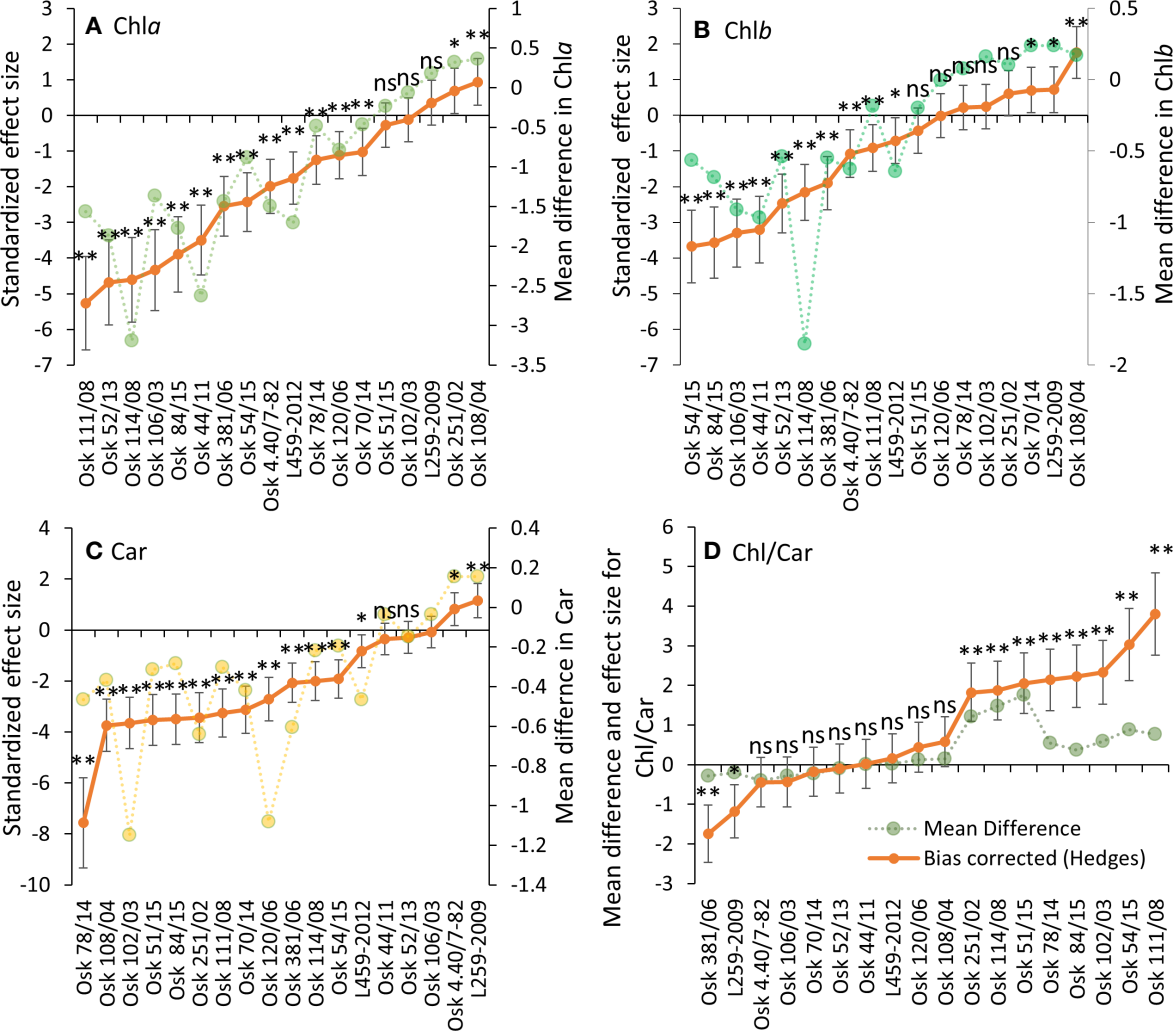
Hedges bias-corrected effect size (with confidence interval) and the mean difference between PEG-induced drought and control treatments for pigment content: Chl*a* **(A)**, Chl*b* **(B)**, Car **(C)**, and their ratio **(D)** in 18 genotypes of wheat seedlings. Significant effects of PEG induced drought are marked with asterisks (* p< 0.01, ** p< 0.001), and ns stands for non-significant (p > 0.05).

Physiological drought induced a significant Chl*a* decrease in most samples. Three genotypes had no change in Chl*a*, while in two genotypes, Chl*a* content increased (**Figure 3A**). A similar trend was determined for Chl*b* and Car content (**Figures 3B, C** **)**. In those genotypes that responded to PEG-induced drought with an increase in pigment content (like L259-2009), it was evident that they had no problems adapting to osmotic stress by maintaining high photosynthetic efficiency, which is an adaptive feature, thus enabling a better tolerance of physiological drought. At the same time, a decreasing Chl-to-Car ratio (**Figure 3D**) can imply some photosynthetic apparatus damage. In those genotypes with decreased pigment content, a lower degree of carotenoid loss also reflects adaptive strategy because of their role in antioxidative defense. Like for PRO and MDA, the Type III sum of squares in two-way ANOVA revealed that genotype variability was the most influential in determining the response of pigment content to physiological drought (**Supplementary Material Table 3**).

### 3.5 Chlorophyll *a* fluorescence

#### 3.5.1 PCA and clustering

Up to now, the results showed that drought treatment was the most influential variable affecting plant growth and relative water content, while genotype variability determined with what intensity varieties of winter wheat seedlings responded to drought. In some cultivars, mild drought stress doesn’t simply trigger acclimation to new conditions but results in various degrees of damage. Therefore, chlorophyll *a* fluorescence measurements were used to obtain parameters regarded as indicators of photosynthetic efficiency that could be associated with the damage to the photosynthetic apparatus. A summary of correlations (**Supplementary Material Tables 4–6**) of all measured parameters considering all varieties and treatments (control and physiological drought) allows identification of potential structures in the matrix and quick detection of correlations of interest. Given the extensive range of data, separate and individual correlations were not explained. The results show that many data are in a complex interrelationship, so to provide a complete picture of linear connectivity, data were summarized in a smaller number of components by multivariate analyses.

**Figure 4:**
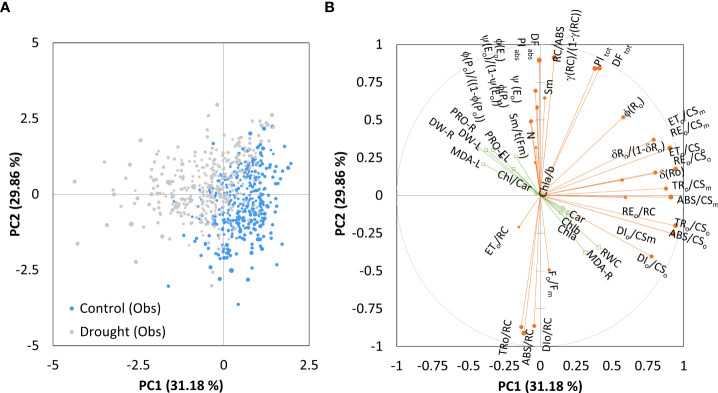
Ordination of all observations **(A)** and correlation between variables on the first two main components **(B)** obtained by principal component analysis on measured parameters (chlorophyll *a* fluorescence as active variables and biochemical measurements as supplementary variables) for control and PEG-induced drought treatment of 18 winter wheat cultivars (n = 720).

**Figure 4** shows the Principal component analysis of the combined chlorophyll *a* fluorescence and biochemical parameters obtained considering control and treatment together. The Kaiser-Meyer-Olkin measure of sampling adequacy was 0.66, and three principal components were distinguished, explaining the variance in 80.4% of the total data. However, complex variables contributing to correlation among both dimensions made them challenging to interpret. After rotation, the first principal component (PC1) accounted for 31.2% of the variance, and the second (PC2) for 29.9%. Positive loadings that characterized the first component with 81.3% contribution were: ABS/CS_o_, DI_o_/CS_o_, TR_o_/CS_o_, ET_o_/CS_o_, RE_o_/CS_o_, ABS/CS_m_, DI_o_/CS_m_, TR_o_/CS_m_, ET_o_/CS_m_, RE_o_/CS_m_, RE_o_/RC, δR_o_, δR_o_/1-δR_o_, representing phenomenological energy fluxes per cross-section of PSII and the efficiency with which an electron is transferred to final PSI acceptors. Given the position of the control and PEG-treated samples along the PC1axis, the PEG-induced drought has changed the phenomenological energy flows to some extent and affected the transport of electrons to the end receptors (**Figure 4A**).

The PC2 was characterized by positive loadings with 62.8% contribution, which were related to the efficiency of the water-splitting complex and the density of active reaction centers at the donor side of PSII (φP_0_/1-φP_0,_ γRC/1-γRC), maximum quantum yield (φP_0_), the quantum yield of photoinduced electron transport at the acceptor side of PSII (ψE_0_, φE_0_), the pool size of electron carriers (S_M_, N) and the performance indices on absorption basis (PI_ABS_). Negative loadings contributed 24% and included adsorbed photon flux by the antenna of PSII units, the part of trapped photon flow by PSII active units that leads to Q_A_ reduction, and the amount dissipated in the PSII antenna (ABS/RC, TR_o_/RC, and DI_o_/RC). When all samples were considered, biochemical measurements had a low contribution to all axes. RWC had the highest positive loading to PC1 (0.408), while negative loadings to PC1 were determined for root dry weight (-0.443) and leaf MDA (-0.397). All others were lower than that and contributed to the complexity, thus preventing differentiation between components.

**Figure 5:**
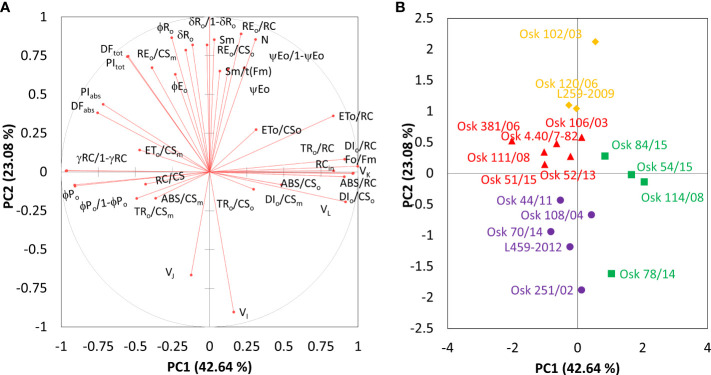
Principal component analysis of chlorophyll *a* fluorescence parameters derived as the mean difference between PEG-induced drought treatment and control samples for 18 winter wheat cultivars. Correlations between obtained parameters and the two principal components **(A)** and projections of genotypes **(B)** with only centroids that are displayed and sorted by hierarchical cluster analysis (• Cluster 1, ◼ Cluster 2, ▲ Cluster 3, ♦ Cluster 4).

To further evaluate and compare the magnitude of cultivar diversity among tested winter wheat seedlings in response to imposed physiological drought, principal component analysis was repeated on the results of the difference test between treatment and control samples. The main components (PC) scores explaining more than 80% variance in the data were then used as input variables for hierarchical cluster analysis (HAC) to classify wheat cultivars’ entries based on their similarity and dissimilarity response. **Figure 5** presents the chlorophyll *a* fluorescence parameters distribution on the first two principal components and locations of observed genotypes as centroids of tested data. Factor loadings after oblimin rotation differentiated three main components explaining 82.2% of the total variance in the data (**Table 3**). Significant positive loadings contributed to the first principal component with 83.4% contribution. They included parameters connected to the dissipation mechanisms (ABS/RC, DI_0_/RC, DI_0_/CS_0_), parameters related to the disconnection of the tripartite system (RC-core antenna-LHC) described by variable fluorescence at L-band (V_L_), inactivation of the oxygen-evolving system described by variable fluorescence at K-band (V_K_), trapped photon flow and flow of electrons transferred from Q_A_^-^ to PQ per active PSII (, and simultaneously, negative loadings of the efficiency of the water-splitting complex and the density of active reaction centers at the donor side of PSII (φP_0_/1-φP_0_, γRC/1-γRC) as well as a maximum efficiency of PSII photochemistry (φP_0_) and performance index on absorption basis (PI_ABS_).

**Table 3:** Correlations between variables and factors and variable contribution (%) after oblimin rotation.

	Correlations			Contribution (%)		
	PC1	PC2	PC3	PC1	PC2	PC3
F_o_/F_m_	**0.907**	-0.023	-0.176	5.266	0.068	0.182
V_L_	**0.936**	-0.302	-0.035	5.331	0.359	0.009
V_K_	**0.905**	-0.14	0.009	5.226	0.009	0.077
V_J_	-0.056	***-0.653***	0.187	0.099	4.307	0.394
V_I_	0.277	**-0.92**	-0.084	0.167	7.965	0.135
Sm	-0.061	**0.855**	-0.189	0.006	7.145	0.413
N	0.217	**0.822**	-0.199	0.606	7.153	0.37
S_m_/t(F_m_)	0.004	***0.648***	-0.21	0.03	4.15	0.536
ABS/RC	**0.965**	-0.123	-0.032	5.953	0	0.019
DI_o_/RC	**0.995**	-0.081	-0.106	6.335	0.014	0.019
TR_o_/RC	**0.905**	-0.14	0.009	5.226	0.009	0.077
ET_o_/RC	**0.792**	0.264	-0.117	4.407	1.283	0.037
RE_o_/RC	0.095	**0.863**	0.105	0.286	7.773	0.297
Φ(P_o_)	**-0.907**	0.023	0.176	5.266	0.068	0.182
Ψ(E_o_)	0.056	***0.653***	-0.187	0.099	4.307	0.394
Φ(E_o_)	-0.299	***0.661***	-0.116	0.342	3.891	0.203
δ(R_o_)	-0.268	**0.802**	0.162	0.167	6.048	0.431
Φ(R_o_)	-0.367	**0.895**	0.103	0.418	7.355	0.168
ABS/CS_o_	0.432	-0.16	**0.843**	1.487	0.064	11.457
DI_o_/CS_o_	**0.800**	-0.103	0.428	4.436	0.001	3.544
TR_o_/CS_o_	0.242	-0.169	**0.936**	0.556	0.119	13.62
ET_o_/CS_o_	0.222	0.216	**0.795**	0.623	0.732	10.145
RE_o_/CS_o_	-0.152	**0.811**	0.487	0.002	6.592	3.821
ABS/CS_m_	-0.405	-0.147	**0.903**	0.844	0.279	11.415
DI_o_/CS_m_	0.432	-0.16	**0.843**	1.487	0.064	11.457
TR_o_/CS_m_	***-0.527***	-0.13	**0.830**	1.544	0.283	9.37
ET_o_/CS_m_	***-0.541***	0.181	**0.763**	1.424	0.197	8.055
RE_o_/CS_m_	***-0.503***	**0.708**	0.492	0.963	4.433	3.472
RC/CS	-0.476	-0.045	**0.795**	1.188	0.06	8.709
PI_ABS_	**-0.770**	***0.523***	0.038	3.281	1.867	0
PI_TOT_	***-0.644***	**0.812**	0.089	1.922	5.461	0.07
DF_ABS_	**-0.798**	0.474	-0.011	3.64	1.429	0.045
DF_TOT_	***-0.651***	**0.809**	0.108	1.963	5.424	0.113
γRC/1-γRC	**-0.967**	0.124	0.037	5.968	0.001	0.014
ΦP_o_/1-ΦP_o_	**-0.904**	0.016	0.168	5.247	0.08	0.156
ΨE_o_/1-ΨE_o_	0.158	***0.648***	-0.127	0.342	4.43	0.135
δR_o_/1-δR_o_	-0.226	**0.83**	0.156	0.085	6.58	0.422

Bold red values represent strong correlations (> 0.7) and italic bold moderate correlations (> 0.5). A green (maximal) – yellow (minimal) color scale is applied to visualize variable contribution.

The second principal component (PC2) included positive loadings with a 93.0% contribution related to the pool size of electron carriers (S_m_, N), reduction of end electron acceptors (δR_o_, φR_o_, RE_o_/RC, RE_o_/CS_o_, RE_o_/CS_m_, δR_o_/1-δR_o_) that characterize IP-phase, as well as negative loadings related to variable fluorescence at I-step. All these parameters strongly correlated with PC2 and influenced the total performance on an absorption basis or the whole linear electron transport (PI_TOT_). Moderate correlations included the quantum yield of photoinduced electron transport at the acceptor side of PSII (ψE_0_, φE_0_) and the ability to maintain an electron chain between two photosystems (ψE_0_/1-ψE_0_) on the positive side of the PC2 axis. In contrast, variable fluorescence at J-step (V_J_) was on the opposing side.

And the third principal component (PC3) was related to the density of reaction centers (RC/CS) and the phenomenological energy fluxes per excited cross-section of PSII, the absorbed photon flux (ABS/CS_o_, ABS/CS_m_), maximum trapped photon flux (TR_o_/CS_o_, TR_o_/CS_m_), and the flux of electrons from Q_A_^-^ to PQ pool (ET_o_/CS_o_, ET_o_/CS_m_) per cross-section of PSII, all of which accounted for 88.1% contribution (**Table 3**).

Hierarchical k-means clustering on main components separated investigated genotypes into four distinctive groups (**Supplementary Material Figure 1**) defined by their response to the physiological drought. The observation plot (**Figure 5B**) allowed exploring the correlations between PCs and investigated genotypes. The main advantage of this process was that each genotype was assigned to only one group reflecting the significance of the most important contributors to the total variance at each axis, thus enabling the selection of relevant parameters for classifying genotypes with similar traits. *Cluster 1* is represented by Osk 251/02, 108/04, 44/11, 70,14, and L459-2012; *Cluster 2* by Osk 54/15, 114/08, 84/15, and 78/14; *Cluster 3* by Osk 111/08, 51/15, 381/06, 4.40/7-82, 106/03, and 52/13; and *Cluster 4* by Osk 102/03, 120/06, and L259-2009. Clusters of winter wheat genotypes will be described with a few of the most frequently used damage indicators derived from chlorophyll *a* fluorescence measurements.

#### 3.5.2 Stability of oxygen-evolving complex, energetic connectivity, and photosynthetic efficiency indices

**Figure 6:**
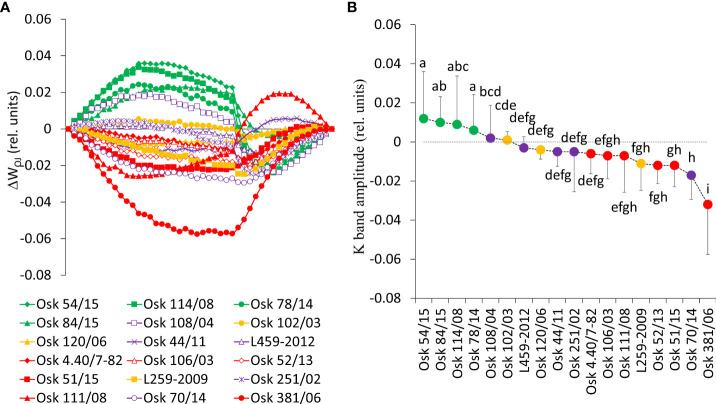
**(A)** Differential curves of relative variable fluorescence O-J normalized induction curves for eighteen wheat genotypes as W_OJ_ = (F_t_-F_o_)/(F_J_-F_o_), each line averages 20 measurements. For the analysis of different kinetics and to reveal the band (K-band) that is typically hidden between steps O and J, the divergences between the relative variable fluorescence curves of the stress treatment (PEG induced water deficit, Ψ = -0.33 MPa) and control (field conditions, water potential Ψ = -0.03 MPa) were calculated as ΔW_OJ_ = W_Treatment_ – W_Control_. **(B)** Mean values of K-band divergences (dispersion refers to maximal values or amplitudes) and statistical differences among genotypes (one-way ANOVA F_17,810_ = 37.3, p< 0.001, values followed by a common letter are not significantly different by the Tukey HSD-test at the 5% level of significance).

A closer look into the earliest phases of the photosynthetic induction curve is presented in the form of differential curves of relative variable fluorescence O-J and O-K normalized induction curves (**Figures 6** **and** **7**). Positive inflections of K-band in genotypes of *Cluster 2* (Osk 54/15, 114/08, 84/15, and 78/14) suggested inhibition of electron flow from the acceptor side of PSII, indicating low OEC activity (**Figure 6**). These genotypes are in the group of drought-susceptible genotypes showing more significant damage to their OEC. Negative deviations of K-band in genotypes of *Cluster 3* indicated that they possess a potential to cope with stress due to their higher stability of OEC and electron transport from PSII to PSI for driving energy synthesis. Genotypes with suppressed K-band (*Cluster 1 and 4*) indicated enhanced resistance of PSII to PEG-induced physiological drought since they can resist drought-induced imbalance in electron flow at the acceptor and donor sides of PSII.

**Figure 7:**
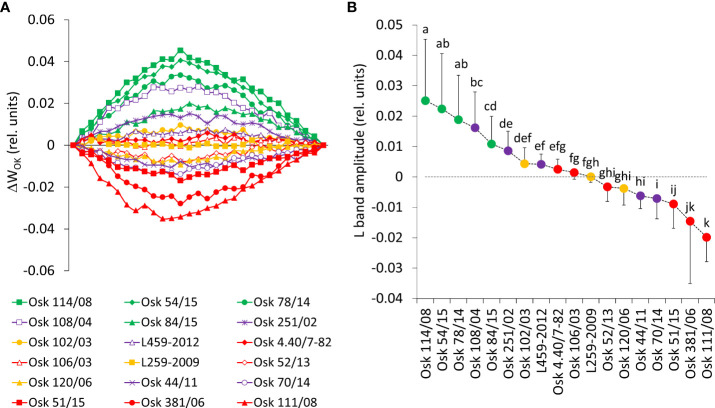
**(A)** Differential curves of relative variable fluorescence O-K normalized induction curves for 18 wheat genotypes as W_OK_ = (F_t_-F_o_)/(F_K_-F_o_), each line averages 20 measurements. For the analysis of different kinetics and to reveal the band (L-band) that is typically hidden between steps O and K, the divergences between the relative variable fluorescence curves of the stress treatment (PEG induced water deficit, Ψ = -0.33 MPa) and control (field conditions, water potential Ψ = -0.03 MPa) were calculated as ΔW_OK_ = W_Treatment_ – W_Control_. **(B)** Mean values of L-band divergences (dispersion refers to maximal values or the amplitude) and statistical differences among genotypes (one-way ANOVA F_17,522_ = 87.7, p< 0.0001, means followed by a common letter are not significantly different by the Tukey HSD-test at the 5% level of significance).

Results shown in **Figure 7** demonstrate that, in susceptible wheat genotypes, even mild drought stress caused a distinct decrease of the energetic connectivity with positive L-bands determined in genotypes of *Cluster 2*, based on the loss of OEC functionality or loss of stability in the tripartite system that controls the first stage of light-harvesting or the LHC-core antenna-RC complex. On the other hand, negative deviations of the L-band indicated an increase in cooperativity of excitation energy exchange between PSII units upon PEG treatment, thus resulting in more efficient consumption of the excitation energy and higher stability of the photosynthetic system (in lines of *Cluster 3*: Osk 111/08, 381/06, 51/15). In genotypes with the observed marginal change of L-band amplitude (*Cluster 1 and 4*), energy connectivity was maintained since the dissociation of LHCII from the PSII complex was prevented.

**Figure 8:**
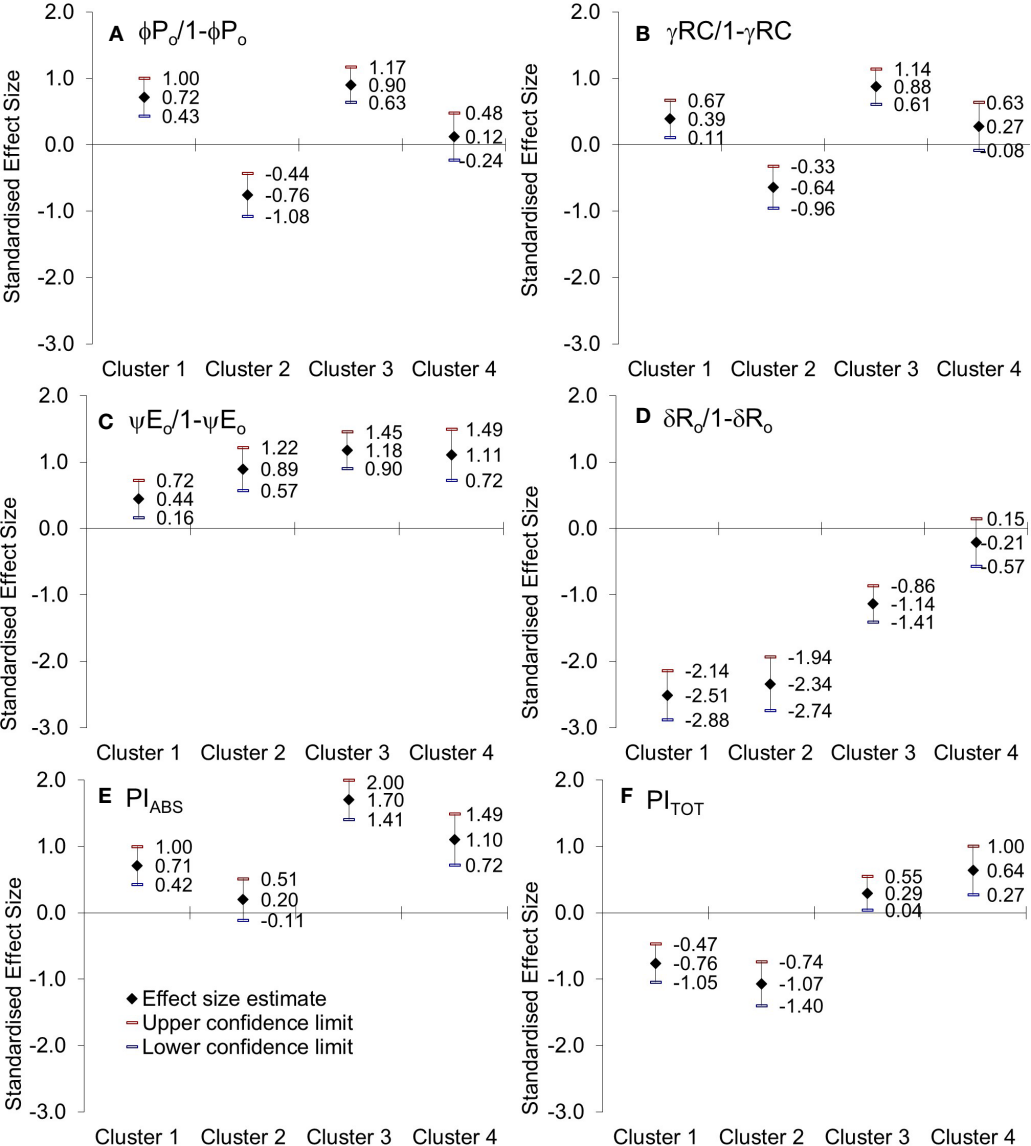
Hedges bias-corrected effect size (with confidence interval) of PEG-induced drought on **(A)** the efficiency of the water-splitting complex, **(B)** the density of active reaction centers at the donor side of PSII, **(C)** the ability to maintain an electron chain between two photosystems, and **(D)** reduction of end electron acceptors (φP0/1-φP0, γRC/1-γRC, ψE_0_/1-ψE_0_, δR_o_/1-δR_o_)), **(E)** performance index on absorption basis (PI_ABS_) and **(F)** performance index (potential) for energy conservation from exciton to the reduction of PSI end acceptors (PI_TOT_) in four obtained Clusters of winter wheat genotypes.

Since the calculated PIs values ​​are relative, they alone cannot be used to characterize samples. What is significant are the changes that occur in PI_ABS_ and PI_TOT_ following any environmental disturbance or stress on the photosynthetic tissue. **Figure 8** presents the estimates of the difference between control and treatment samples and shows variations in the response of genotype clusters to imposed physiological drought, which will be elaborated further in the discussion part.

## 4 Discussion

Cell membrane stability appears to be a valuable preliminary method for screening wheat seedlings for drought susceptibility since the cell membrane is the primary site of damage under stress. The decrease in cell membrane integrity was evident in all genotypes, probably as a result of overproduction of H_2_O_2_ (Naderi et al., 2020), which not only causes changes in the composition of membrane proteins and lipids as evidenced by the content of MDA but also plays a signaling role and stimulates the synthesis of osmolytes and antioxidant enzymes that participate in the defense against oxidative stress (Singh et al., 2012). However, little phenotypic variability was noticeable in PEG-test since the differences were significant only between genotypes with the lowest and the highest cell membrane integrity. Therefore, moderate drought stress was reduced to mild, within physiological limits, because minor water deficit most likely results in lesser or greater acclimation to drought depending on plants’ natural and inherent characteristics. Consequently, many changes occur in the structure and physiology of plants due to stress caused even by mild physiological drought. The results show that PEG-induced drought was the most influential variable that affected plant growth since shoot height was reduced in all genotypes and root length in most of them. In all genotypes, the whole plant underwent anatomical and morphological changes to prevent metabolic imbalance and maintain the content and transport of water. Since plants often show phenotypic plasticity to minimize the adverse effects of environmental stressors (Grenier et al., 2016), genotypes that invest in the root system (like L459-2012 and Osk 70/14) are considered drought resistant (Liu et al., 2015). These differences in shoot and root growth were also reflected in their ratio. It is well known that as the plant ages, the root-to-shoot ratio decreases, showing priority to collecting light energy. However, in arid conditions, the increased root-to-shoot ratio indicates increased root growth that will provide plants with access to water (El Siddig et al., 2013). Therefore, a higher root-to-shoot ratio is essential when choosing drought-resistant varieties. Significant inter-genotypic differences were also observed in the dry matter accumulation among wheat varieties both in the root and in the shoot. Also, the translocation of a relatively higher percentage of dry weight was observed towards the root system when exposed to PEG-induced drought (the most pronounced in genotypes Osk 70/14, Osk 381/06, and Osk 84/15). According to some authors (Carvalho et al., 2014), this indicates a better redistribution of accumulated carbon to the plant’s root system and is an adaptive trait.

The evident water retention in all wheat genotypes resulted from effective water use, suggesting that all wheat seedlings’ genotypes acclimated to imposed physiological drought to avoid dehydration (Sial et al., 2017). This was also visible in a common adaptive feature of leaf rolling in response to water deficit, reducing transpiration and water use. It is also possible that the osmotic adjustment contributed to maintaining a high relative water content (Silva et al., 2010) since it facilitates turgor maintenance by lowering the osmotic potential of the cell (Blum, 2017). However, osmotic adjustment can lead to anomalously low estimates of relative water content (Boyer et al., 2008). The activation of the metabolic pathways responsible for the synthesis of proline under conditions of mild stress suggested that all genotypes have possibilities for preventing adverse effects of imposed drought (Bandurska et al., 2017). Despite extensive research on proline accumulation under water deficit conditions, there are still conflicting opinions on the correlation between proline content and drought resistance. Some authors believe that increased proline content in plant tissues results from dehydration and is associated with sensitivity to drought (Schafleitner et al., 2007; Nazar et al., 2015; Blum, 2017; Mu et al., 2021). However, research on cereals such as barley and wheat shows that increased proline content is a feature of stress-tolerant varieties (Sultan et al., 2012; Ahmed et al., 2013). Nevertheless, the importance of proline accumulation for adaptation to drought is still uncertain. What is certain is that proline is a “compatible” solute that contributes to the osmotic adjustment of the cytoplasm (Blum, 2017). Decreased MDA levels in roots as opposed to increased MDA levels in leaves under mild drought stress can also be explained by the synthesis of osmolytes (Sultan et al., 2012) and indicate a higher antioxidant ability, which contributes to better drought resistance of wheat seedlings (Dhanda et al., 2004; Shao et al., 2005). However, one has to bear in mind that the primary sites of reactive oxygen accumulation are the plant leaves (Sahu and Kar, 2018; Liu et al., 2022), which also correlates well with higher carotenoid content in the same genotypes that reduce reactive oxygen species and inhibit lipid peroxidation (Shao et al., 2008; Jaleel et al., 2009). Overall, results show that biochemical and physiological responses to mild drought stress depend on the genetic predispositions of each variety, which has also been established in other plant species such as sesame (Fazeli et al., 2007), rice (Shobbar et al., 2010), cherries (Medeiros et al., 2012) and thyme (Bahreininejad, 2013).

Since photosynthesis is one of the plants’ most essential and sensitive processes that any minor stressful event can disrupt, the best way to investigate changes in the functioning and the structure of the photosynthetic apparatus under imposed drought conditions is by fast, non-destructive, and relatively simple chlorophyll *a* fluorescence technique (Strasser and Strasser, 1995; Strasser et al., 2004a; Tsimilli-Michael and Strasser, 2013; Dąbrowski et al., 2016; Goltsev et al., 2016; Kalaji et al., 2018). To find directions that best explain the variance in the data sets, a Principal Component Analysis reduced chlorophyll *a* fluorescence parameters to a group of most informative ones (Goltsev et al., 2012). Accordingly, control samples are characterized by suitable phenomenological energy fluxes per cross-section of PSII and the efficiency with which an electron is transferred to final PSI acceptors. At the same time, PEG-induced drought changed, to some extent, phenomenological energy fluxes, and the significant influence was on electron transport to end receptors. Similar responses were described in barley (Oukarroum et al., 2009), wheat (Brestic et al., 2012), rice (Wang et al., 2017), and *Tilia cordata* Mill (Kalaji et al., 2018).

The second direction that could explain variance in the results relates to the efficiency of the water-splitting complex and the density of active reaction centers at the donor side of PSII, the maximum quantum yield and the performance indices on an absorption basis, as well as absorbed photon flux, the part of trapped and the amount dissipated photon flow in the PSII antenna. However, considering the whole data set, this direction cannot be used to discern PEG-induced drought from the control samples due to high variability in winter wheat cultivars’ response to imposed conditions. Therefore, PCA of the mean difference test data (by comparing the impact) and subsequent HAC analysis enabled trade-offs among chlorophyll *a* fluorescence parameters and revealed clustering of relevant parameters and genotypes based on their response to imposed physiological drought. Three groups of relevant fluorescence parameters were determined. The first, PC1, was characterized by photochemical parameters representing the donor and acceptor side of PSII. The second, PC2, is defined by the parameters of the thermal phase of the photosynthetic process and the acceptor side of PSI, representing the electron flow around PSI and the chain of electrons between PSII and PSI. While the third component, PC3, consisted of phenomenological energy fluxes per cross-section. This grouping of fluorescence parameters more accurately separated the investigated genotypes into four distinct clusters based on their response to imposed physiological drought conditions. It enabled an explanation of the specificity of their reaction.

### 4.1 Classification of winter wheat genotypes and their associated characteristics

Genotypes of *Cluster 1* were well correlated with PC2, characterized by an increase in variable fluorescence at the I-step and a related decrease in the reduction of end electron acceptors that influenced the total performance (PI_TOT_), **Figure 8** and **Supplementary Material Figure 2**. They were also characterized by no change in free proline, MDA levels, or relative water content. The suppressed K-band indicated enhanced resistance of PSII to PEG-induced physiological drought, meaning that they can resist drought-induced imbalance in electron flow at the acceptor and donor sides of PSII. Similarly, the observed marginal change of L-band amplitude indicated that energy connectivity was maintained since the dissociation of LHCII from the PSII complex was prevented. However, an attenuated L-band still can show a loss in energetic connectivity to some extent, which could be due to reaching the drought acclimation threshold or indicating the presence of drought avoidance mechanisms. Nevertheless, increased phenomenological parameters stimulated an increase in PI_ABS_. Therefore, these genotypes were sensitive to PEG-induced drought but, most probably due to their excellent initial stability and tolerance of photosynthetic apparatus, were able to acclimate. Similar responses were determined in *Arabidopsis thaliana* plants adapted to different light intensities and temperature conditions (Ballottari et al., 2007) and in cold stress-tolerant zoysia grass cultivars (Gururani et al., 2015).

On the other hand, genotypes of *Cluster 2* were characterized by inactivation of reaction centers, disconnection of tripartite system LHC-core antenna-RC, inactivation of oxygen-evolving complex, and increase in dissipation, all leading to the decrease in the first reactions at PSII, that is, a reduction of maximum efficiency of PSII photochemistry, performance on absorption basis (PI_ABS_), as well as variable fluorescence at I-step. A similar decrease was determined in wheat exposed to slowly advancing drought stress in natural conditions (Zivcak et al., 2008a; Zivcak et al., 2008b). Positive inflections of the K-band in these genotypes (Osk 54/15, 114/08, 84/15, and 78/14) suggested inhibition of electron flow from the acceptor side of PSII, indicating lower OEC activity that could lead to an incomplete water splitting process and result in ROS production undermining photosynthesis, i.e., distracting electron balance between OEC and tyrosine (Guha et al., 2013; Najafpour et al., 2013). Similarly, positive L-bands demonstrated that even mild drought stress caused a distinct decrease in the energetic connectivity based on the loss of OEC functionality or stability in the tripartite system that controls the first stage of light-harvesting or the LHC-core antenna-RC complex (Oukarroum et al., 2007; Zhou et al., 2019). Furthermore, these genotypes had increased free PRO in roots and leaves, although to a minor degree, and MDA level in leaves, all indicative of adjustment to some degree and activation of defense mechanisms. Since *Cluster 2* was characterized by a significant reduction in the RC/ABS parameter and correlated well with PC1 having moderate to low initial stability and, therefore, high sensitivity due to a considerable impact on photosynthetic apparatus results in their lower potential to cope with stress.

*Cluster 3* genotypes’ responses were best described by PC1 and PC3. Their phenomenological energy fluxes per cross-section (ABS, TR, and ET per CS_o_ and CS_m_) were significantly decreased by PEG-induced drought. Reduced phenomenological parameters could indicate a negative influence of imposed drought at the early stages of its action. At the same time, the fraction of active reaction centers increased after stress. With its negative deviations, K-band indicated the better potential of these lines to cope with stress due to higher stability of OEC and electron transport from PSII to PSI for driving energy synthesis. Likewise, negative deviations of the L-band indicated an increase in cooperativity of excitation energy exchange between PSII units upon PEG treatment, thus resulting in more efficient consumption of the excitation energy and higher stability of the photosynthetic system (Strasser et al., 2004a). This boosted the first reactions in PSII (photon to exciton trapping events) and enhanced the ability to maintain the electron flow between PSII and PSI, thus increasing the driving forces of photosynthesis performance and PI_ABS_. However, increased free PRO indicated osmotic adjustment, and the highest MDA level indicated possible oxidative damage. At the same time, carotenoids decreased, most probably because of involvement in the detoxification of reactive oxygen species. Therefore, genotypes of this cluster appeared to be sensitive to physiological drought due to the negative influence on PSI. Still, they successfully acclimated to some point by activation of defense mechanisms.

PEG-induced drought did not affect the energy flux associated with the electron transport from Q_A_^-^ to final acceptors of PSI in genotypes Osk 120/06, 102/03, and L259-2009 that were grouped as *Cluster 4*. In addition, the response of these genotypes was best explained by their increased pool size of reduced PQ (N, S_m_), Q_A_^-^ that is reduced more often, and by increased potential for the reduction of end electron acceptors. The accumulation of reducing equivalents favors cyclic electron transport around PSI, which supplies additional ATP to chloroplasts (Huang et al., 2019; Huang et al., 2021). The acceleration of cyclic electron flow around PSI most probably accelerates repair of PSII activity, allowing these genotypes to perform better in mild stress. These genotypes were characterized by marginal change K- and L-band amplitude indicative of enhanced resistance to a drought-induced imbalance in electron flow at the acceptor and donor sides of PSII and maintained energy connectivity since the dissociation of LHCII from the PSII complex was prevented. Furthermore, these genotypes had the most increased PRO levels indicating an osmotic adjustment; RWC was no different from the control samples, and so were MDA levels in roots and leaves. All these adaptive features point out that these genotypes could resist physiological drought by showing a rapid acclimation of the photosynthetic system and osmotic adjustment, therefore, having a higher potential to cope with stress. As PI_TOT_ reflects the functionality of both photosystems and gives quantitative information about the current status of the plant in stressful conditions (Zivcak et al., 2008b; Živčák et al., 2014; Samborska et al., 2019), an increase in *Cluster* ***4*** implies outstanding functionality of PSI and PSII in mild drought stress conditions. This observed increase in electron transport in the early development of seedlings may be related to the activation of mechanisms responsible for drought tolerance (Kovačević et al., 2017). Similar findings that leave developing in drought conditions, especially under mild stress, increases its photosynthetic efficiency, most probably to compensate and use this to boost metabolism upon recovery are described in several papers (Xu et al., 2009; Avramova et al., 2015; Vincent et al., 2020). One more parameter indicative of drought stress is the IP phase which illustrates an imbalance between Q_A_ reduction and oxidation and the plastoquinone pool. Since the IP phase depends on the efficiency of the PSI acceptor’s electron uptake and the number of available oxidized forms of NADP, the negative values ​​of the IP phase (the data of which are not presented in this paper but can be correlated to V_I_) in genotypes of ***Cluster 4*** corresponded to a larger number of oxidized forms of NADP (NADP+) molecules per active center. This was reflected in the lower need for reductants on the PSI acceptor side (Ceppi et al., 2011; Pollastrini et al., 2014; Kula-Maximenko et al., 2021) and could be a compensatory mechanism for seedlings that have evolved in response to suboptimal environmental conditions.

Since every change in the OJIP curve is reflected in the index of photosynthetic efficiency (PI_TOT_) - an energy conversion from exciton to the reduction of the final electron acceptor in PSI (Zivcak et al., 2014; Kalaji et al., 2016; Kalaji et al., 2017; Tsimilli-Michael, 2020), PI_TOT_ showed to be the most sensitive parameter of the JIP-test in detecting and comparing the intensity of stress effect among tested genotypes. However, the explanation of the seedling’s response inevitably included independent pieces of essential parameters embedded in PIs (as seen in **Figure 8** and **Supplementary Material Figure 2**): the maximum quantum yield of primary photochemistry – φP_o_ (using F_0_ and F_M_), the efficiency of electron transport further from Q_A_^-^ - ψ(E_o_) (using V_J_), the efficiency with which the electron moves from the reduced electron acceptors to the final acceptors - δ(R_o_) (using V_J_ and V_I_) and the ratio of chlorophyll concentration of reaction centers and chlorophyll antennae - RC/ABS (using φP_o_, V_J_ and the initial slope of the OJIP curve). However, can we honestly choose one or two chlorophyll fluorescence parameters to characterize drought tolerance of winter wheat genotypes? It does not seem like it. As stated in the review paper Tsimilli-Michael (2020), comparing the impact of imposed stress (i.e., physiological drought in this paper) on a whole set of parameters enables the identification of specific effects in the electron transport chain. Selecting those that better explain the individual plant’s response gives a significant advantage in screening genotypes if the comparison of stress effect within physiological limits is in question. Multivariate analyses and data mining of all parameters after stress enables the exploration of physiological processes. Nonetheless, these parameters only provide access to mechanisms. At the same time, biochemical and physiological measurements are needed for interpretation, which is proven in this paper.

The genetic contribution to drought adaptation is based on a combination of constituent and induced physiological and biochemical properties. Apprehending interactions of a complex collection of traits that enable acclimation to drought is much more complicated than understanding the functioning of an individual attribute. However, drought acclimation is often the result of a collective expression of many plant characteristics in the appropriate environment. Therefore, to better understand the relative importance of the different mechanisms, it is necessary to research a high number of varieties of the same species. Similarly, understanding the reactions of seedlings at all levels and to all factors affecting them has great value because the developmental stages in the same group generally show close similarities or several confusing differences, especially since the development of specialized adaptive traits has not yet begun. A critical step in conducting such research is developing and improving screening methods for identifying and evaluating functional relationships of relevant characteristics that are useful for acclimation, acclimatization, and adaptation to different types of drought stress and to be able to do it in all essential phenological stages of plant development. Therefore, the long-term vision of research and breeding programs should also include screening methods on seedlings to help identify, characterize, and select crucial phenotypic traits to find genetic markers for specific characteristics that can contribute to adaptation to, e.g., drought.

## 5 Conclusion

PEG-induced physiological drought enabled reliable screening of winter wheat genotypes in the first phase of seedling establishment. Chlorophyll *a* fluorescence appeared to be an effective method of differentiating sensitive and tolerant genotypes. Drought treatment was the most influential variable affecting plant growth and relative water content, while genotype variability determined with what intensity varieties of winter wheat seedlings responded to drought. As for chlorophyll *a* fluorescence parameters, PCA of all datasets showed that PEG-induced lack of water mainly influenced phenomenological energy fluxes and the efficiency with which an electron is transferred to final PSI acceptors. Fluorescence parameters that accurately described tested genotypes based on the effect size were grouped around three major components: photochemical parameters (PC1), representing the donor and acceptor side of PSII; thermal phase of the photosynthetic process and the acceptor side of PSI (PC2), representing the electron flow around PSI and the chain of electrons between PSII and PSI; and phenomenological energy fluxes per cross-section (PC3). The most reliable parameters of the JIP-test in detecting and comparing the drought impact among tested genotypes were variable fluorescence at K, L, and I step and PI_TOT_. Four distinct clusters of genotypes were discerned based on their response to imposed physiological drought, and the integrated analysis of biochemical and physiological parameters explained their reactions’ specificity. Multivariate analyses and data mining of all parameters after stress enabled the exploration of physiological processes in all genotypes, thus complementing the knowledge needed to address fundamental issues, like plasticity, in young and fully developed plants and understand the physiological processes that lead to tolerance.

## Data availability statement

The original contributions presented in the study are included in the article/**Supplementary Material**. Further inquiries can be directed to the corresponding author.

## Author contributions

VP was responsible for conceptualization, methodology, research, formal analysis, supervision, writing of original draft, review & editing. AA and JD were accountable for the study, lab analysis, and data collection. GD and VC provided resources, funding acquisition, critical review & editing of the initial draft. All authors contributed to the article and approved the submitted version.

## Funding

This work was supported by the Department of Biology, Josip Juraj Strossmayer University of Osijek Research Block Grant Program.

## Conflict of interest

The authors declare that the research was conducted in the absence of any commercial or financial relationships that could be construed as a potential conflict of interest.

## Publisher’s note

All claims expressed in this article are solely those of the authors and do not necessarily represent those of their affiliated organizations, or those of the publisher, the editors and the reviewers. Any product that may be evaluated in this article, or claim that may be made by its manufacturer, is not guaranteed or endorsed by the publisher.

## Glossary

**Table:**

ABS	absorbed photon flux
ABS/RC	average absorbed photon flux per PSII reaction center
ABS/CS	absorbed photon flux per excited cross-section of PSII (or also apparent antenna size)
Car	carotenoid
Chl	chlorophyll
CS	a cross-section of PSII
DI_0_/ABS	quantum yield of energy dissipation in PSII antenna
DI_0_/RC	The flux of energy dissipated per active RC
ET_0_/RC	electron transport flux from to PQ per active PSII
F_0_	initial fluorescence value
F_300μs_	fluorescence value at 300 μs
F_m_	maximal fluorescence intensity
F_V_	maximum variable fluorescence
OEC	oxygen-evolving complex
PCA	principal component analysis
PI_ABS_	Performance index (potential) for energy conservation from exciton to the reduction of intersystem electron acceptors
PI_TOT_	Performance index (potential) for energy conservation from exciton to the reduction of PSI end acceptors
Q_A_ and Q_B_	primary and secondary quinone electron acceptor
PQ	the pool of free plastoquinone behind the PSII reaction center
PQH_2_	plastoquinol
PSI	photosystem I
PSII	photosystem II
RC	total number of PSII active reaction centers
RE_0_/RC	electron transport flux from to final PSI acceptors per active PSII
RE_0_/CS	electron transport flux from to final PSI acceptors per cross-section of PSII
S_m_	the normalized area between OJIP curve and the line
F_m_	which is a proxy of the number of electron carriers per electron transport chain
TR_0_/RC	maximum trapped exciton flux per active PSII
TR_0_/CS	maximum trapped exciton flux per cross-section
V_I_	relative variable fluorescence at 30 ms (I-step)
V_J_	relative variable fluorescence at 2 ms (J-step)
V_K_	relative variable fluorescence at 300 μs (K-step)
V_L_	variable fluorescence at L-band
V_t_	relative variable fluorescence at time t
ΔV_IP_	I-P normalized differential induction curves
ΔV_OJ_	O-J normalized differential induction curves
ΔV_OK_	O-K normalized differential induction curves
ΔV_OP_	O-P normalized differential induction curves
δR_0_	efficiency with which an electron from Q_B_ is transferred to final PSI acceptors
φE_0_	Quantum yield of electron transport from to PQ
φP_0_	maximum quantum yield of primary PSII photochemistry
φR_0_	quantum yield of electron transport from to the final PSI acceptors
ψE_0_	efficiency with which a PSII trapped electron is transferred from Q_A_ to Q_B_
ψR_0_	efficiency with which a PSII trapped electron is transferred to final PSI acceptors
