# Supplementary material for: PEG-induced physiological drought for screening winter wheat genotypes sensitivity – integrated biochemical and chlorophyll a fluorescence analysis
Source: Front Plant Sci. 2022 Oct 12;13:987702. doi: 10.3389/fpls.2022.987702 (PMC9597320; doi:10.3389/fpls.2022.987702)
Supplement: Supplementary file 1 [file DataSheet_1.docx]

Supplementary Material

## Supplementary Figures and Tables

**Supplementary Material Table 1.** Summary of measured fast chlorophyll *a* fluorescence transient and calculated OJIP test parameters where O stands for origin (the minimum fluorescence F_0_), J and I are intermediate levels at 2 ms and 30 ms, and P is a peak at F_m_ or maximal fluorescence. PSI – photosystem I, PSII – photosystem II, RC – total number of PSII active reaction centers, CS – a cross-section of PSII, and Q_A_ – plastoquinone A, Q_A_ –plastoquinone B, and PQ – the pool of free plastoquinone behind the PSII reaction center.

| Parameter | Description |
| --- | --- |
| *Technical fluorescence parameters* | |
| $F_{0}\cong F_{0.02ms}$  $F_{m}$  $F_{V}=F_{m}-F_{0}$  $V_{t}={(F_{t}-F_{0})}/{(F_{m}-F_{0})}$  $M_{0}={({\Delta V}/{\Delta t)}}_{0}$  $\approx4(F_{0.3ms}-F_{0.02ms})/F_{V}$  $S_{m}=Area/F_{V}$ | Initial fluorescence value (at 20 μs)  Maximal fluorescence value (at 30 ms)  Maximum variable fluorescence  Relative variable fluorescence  Initial slope (in ms^-1^) of the O-J  The normalized area between the OJIP curve and the line $F_{m}$, which is a proxy of the number of electron carriers per electron transport chain |
| *Energy fluxes* | |
| ABS_0_  TR_0_  DI_0_  ET_0_  RE_0_ | Adsorbed photon flux (rate of photon absorption by total PSII antenna  Trapped exciton flux (rate of exciton trapping by all PSII RCs leading to Q_A_ reduction)  Dissipated energy flux (rate of energy dissipation in all PSII other than trapping)  Electron transport flux from Q_A_ to Q_B_  Electron transport flux until PSI acceptors |
| *Efficiencies and quantum yield* | |
| ${\psi E}_{0}= {ET}_{0}/{TR}_{0}$  ${\psi R}_{0}= {RE}_{0}/{TR}_{0}$  ${\delta R}_{0}= {\psi R}_{0}/{\psi E}_{0}$  ${\varphi P}_{0}= {TR}_{0}/ABS$  ${\varphi E}_{0}= {\varphi P}_{0}\times{\psi E}_{0}$  ${\varphi R}_{0}= {\varphi P}_{0}\times{\psi R}_{0}$  ${DI}_{0}/ABS$  $RC/ABS$ | Efficiency with which a PSII trapped electron is transferred from Q_A_ to Q_B_  Efficiency with which a PSII trapped electron is transferred to final PSI acceptors  Efficiency with which an electron from Q_B_ (PQH_2_) is transferred to final PSI acceptors  The maximum quantum yield of primary PSII photochemistry  Quantum yield of electron transport from $Q_{A}^{-}$ to PQ  Quantum yield of electron transport from $Q_{A}^{-}$ to final PSI acceptors  Quantum yield of energy dissipation in PSII antenna  Density of PSII RC per antenna chlorophyll (per absorption) |
| *Specific energy fluxes* | |
| $ABS/RC$  ${TR}_{0}/RC$  ${ET}_{0}/RC$  ${RE}_{0}/RC$  ${DI}_{0}/RC$ | Average absorbed photon flux per PSII reaction center (apparent antenna size of an active RC)  Maximum trapped exciton flux per active PSII  Electron transport flux from $Q_{A}^{-}$ to PQ per active PSII  Electron transport flux from $Q_{A}^{-}$to final PSI acceptors per active PSII  The flux of energy dissipated (other than trapping) per active PSII |
| *Phenomenological energy fluxes (per excited cross-section CS)* | |
| $ABS/{CS}_{0}\approx F_{0}$and $ABS/{CS}_{m}\approx F_{m}$  ${TR}_{0}/CS$  ${ET}_{0}/CS$  ${RE}_{0}/CS$ | Absorbed photon flux per excited cross-section of PSII (or also apparent antenna size)  Maximum trapped exciton flux per cross-section  Electron transport flux from $Q_{A}^{-}$ to PQ per cross-section of PSII  Electron transport flux from $Q_{A}^{-}$ to final PSI acceptors per cross-section of PSII |
| *Performance indexes (of PSII and specific electron transport reactions)* | |
| ${PI}_{ABS}= {RC}/{ABS\times}\left[ {\varphi P}_{0}/\left( 1-{\varphi P}_{0} \right) \right] \times\left[ {\psi E}_{0}/\left( 1-{\psi E}_{0} \right) \right]$  ${PI}_{ABS}^{total}={PI}_{ABS}\times\left[ {\delta R}_{0}/\left( 1-{\delta R}_{0} \right) \right]$ | Performance index (potential) for energy conservation from exciton to the reduction of intersystem electron acceptors  Performance index (potential) for energy conservation from exciton to the reduction of PSI end acceptors |

**Supplementary Material Table 2.** Summary of dry weight percentage in roots and leaves of investigated 18 winter wheat genotypes exposed to control (CON, ψ = -0.033 MPa) and drought-induced stress (PEG, ψ = -0.301 MPa) by PEG. Results are given as mean values ± standard deviation (n = 6) and % increase in DW due to treatment.

|  | % DW - Roots | | |  | % DW - Leaves | | |
| --- | --- | --- | --- | --- | --- | --- | --- |
| Genotype | CON | PEG | % increase |  | CON | PEG | % increase |
| Osk 52/13 | 12 ± 1.0 | 20 ± 1.9 | 68 % |  | 16 ± 0.3 | 18 ± 1.2 | 11 % |
| Osk 106/03 | 11 ± 0.2 | 22 ± 0.9 | 96 % |  | 16 ± 0.6 | 18 ± 1.6 | 15 % |
| Osk 114/08 | 11 ± 0.2 | 20 ± 3.3 | 77 % |  | 16 ± 1.5 | 19 ± 0.2 | 17 % |
| Osk 111/08 | 13 ± 0.6 | 22 ± 1.7 | 67% |  | 16 ± 2.1 | 20 ± 0.5 | 20 % |
| Osk 108/04 | 13 ± 0.5 | 22 ± 1.8 | 65 % |  | 15 ± 0.3 | 19 ± 0.5 | 21 % |
| Osk 4.40/7-82 | 12 ± 0.4 | 22 ± 6.3 | 93 % |  | 14 ± 0.7 | 17 ± 1.0 | 21 % |
| Osk 54/15 | 10 ± 0.2 | 18 ± 0.4 | 78 % |  | 13 ± 0.7 | 16 ± 1.1 | 22 % |
| L459-2012 | 13 ± 0.6 | 23 ± 0.8 | 80 % |  | 15 ± 0.6 | 19 ± 0.7 | 24 % |
| Osk 78/14 | 13 ± 1.5 | 20 ± 1.8 | 61 % |  | 15 ± 0.5 | 18 ± 0.8 | 25 % |
| L259-2009 | 11 ± 0.5 | 21 ± 1.6 | 92 % |  | 16 ± 0.3 | 20 ± 0.3 | 25 % |
| Osk 44/11 | 12 ± 0.4 | 22 ± 2.8 | 79 % |  | 14 ± 0.2 | 18 ± 1.2 | 26 % |
| Osk 381/06 | 14 ± 2.1 | 29 ± 6.6 | 105 % |  | 16 ± 0.9 | 21 ± 2.7 | 26 % |
| Osk 84/15 | 18 ± 4.8 | 31 ± 5.4 | 69 % |  | 15 ± 0.7 | 19 ± 2.0 | 28 % |
| Osk 120/04 | 13 ± 1.7 | 20 ± 1.6 | 61 % |  | 14 ± 0.8 | 17 ± 1.7 | 28 % |
| Osk 251/02 | 14 ± 0.4 | 23 ± 1.4 | 64 % |  | 16 ± 0.1 | 20 ± 1.5 | 30 % |
| Osk 51/15 | 13 ± 1.4 | 21 ± 0.9 | 60 % |  | 16 ± 0.2 | 20 ± 0.5 | 30 % |
| Osk 102/03 | 14 ± 0.9 | 22 ± 1.6 | 56 % |  | 15 ± 0.6 | 20 ± 1.0 | 34 % |
| Osk 70/14 | 12 ± 1.0 | 26 ± 3.5 | 121 % |  | 15 ± 0.1 | 21 ± 1.3 | 41 % |

**Supplementary Material Table 3.** Summary of two-way ANOVA for pigment content, relative water content, malondialdehyde and free proline in roots and leaves of investigated 18 winter wheat genotypes exposed to control (ψ = -0.033 MPa) and drought-induced stress (ψ = -0.301 MPa) by PEG.

|  | | Chl*a* | Chl*b* | Car | Chl*a/b* | Chl/Car | RWC | EL | MDA-Leaf | PRO-Leaf | MDA-Root | PRO-Root |
| --- | --- | --- | --- | --- | --- | --- | --- | --- | --- | --- | --- | --- |
| R² | | 0.860 | 0.540 | 0.657 | 0.458 | 0.748 | 0.909 | 0.692 | 0.853 | 0.997 | 0.842 | 0.995 |
| F | | 120.43 | 22.96 | 37.35 | 16.49 | 58.05 | 194.82 | 44.01 | 113.18 | 5800.09 | 104.02 | 4091.53 |
| Pr > F | | <0.0001 | <0.0001 | <0.0001 | <0.0001 | <0.0001 | <0.0001 | <0.0001 | <0.0001 | <0.0001 | <0.0001 | <0.0001 |
| Genotype (G) | SS | 609.58 | 87.59 | 42.50 | 21.55 | 196.61 | 1.56 | 4432.0 | 989882.0 | 62280.0 | 61393.2 | 87232.4 |
|  | F | 110.525 | 24.702 | 35.519 | 17.244 | 34.228 | 294.002 | 36.787 | 140.278 | 6457.397 | 112.240 | 5501.890 |
|  | Pr > F | <0.0001 | <0.0001 | <0.0001 | <0.0001 | <0.0001 | <0.0001 | <0.0001 | <0.0001 | <0.0001 | <0.0001 | <0.0001 |
| Treatment (T) | SS | 26.395 | 3.029 | 2.565 | 0.016 | 4.483 | 0.004 | 408.0 | 41936.0 | 7825.7 | 24.29 | 78.39 |
|  | F | 81.358 | 14.523 | 36.452 | 0.222 | 13.268 | 12.079 | 57.610 | 101.027 | 13793.648 | 0.755 | 84.057 |
|  | Pr > F | <0.0001 | 0.000 | <0.0001 | 0.637 | 0.000 | 0.001 | <0.0001 | <0.0001 | <0.0001 | 0.385 | <0.0001 |
| G × T | SS | 731.5 | 76.9 | 46.9 | 20.8 | 485.4 | 0.564 | 6077 | 612466 | 45065.9 | 55726.7 | 46247.4 |
|  | F | 132.64 | 21.71 | 39.24 | 16.70 | 84.51 | 106.38 | 50.43 | 86.79 | 4672.58 | 101.88 | 2916.90 |
|  | Pr > F | <0.0001 | <0.0001 | <0.0001 | <0.0001 | <0.0001 | <0.0001 | <0.0001 | <0.0001 | <0.0001 | <0.0001 | <0.0001 |

**Supplementary Material Table 4.** Pearson correlation matrix of morphological and biochemical parameters. The display option used is blue-red, which presents a negative correlation with cold colors (blue for correlations close to -1) and positive correlations with warm colors (red for correlations close to 1). Values in bold are different from 0 with significance level α=0.05; ns stands for non-significant, * p < 0.05, ** p < 0.01, *** p < 0.001.

| **Variables** | **Shoot** | **Root** | **Chl*a*** | **Chl*b*** | **Car** | **Chl(*a*/*b*)** | **Chl/Car** | **RWC** | **EL** | **MDA Leaf** | **MDA Root** | **DW Leaf** | **DW Root** | **PRO Leaf** |
| --- | --- | --- | --- | --- | --- | --- | --- | --- | --- | --- | --- | --- | --- | --- |
| **Root** | **0.779** |  |  |  |  |  |  |  |  |  |  |  |  |  |
| **Chl*a*** | **0.342** | **0.372** |  |  |  |  |  |  |  |  |  |  |  |  |
| **Chl*b*** | **0.345** | 0.279 | **0.630** |  |  |  |  |  |  |  |  |  |  |  |
| **Car** | **0.397** | 0.306 | 0.146 | **0.683** |  |  |  |  |  |  |  |  |  |  |
| **Chl(*a*/*b*)** | 0.004 | 0.098 | **0.505** | **-0.338** | **-0.544** |  |  |  |  |  |  |  |  |  |
| **Chl/Car** | -0.160 | -0.049 | **0.517** | -0.067 | **-0.730** | **0.669** |  |  |  |  |  |  |  |  |
| **RWC** | **0.712** | **0.577** | **0.533** | **0.551** | **0.514** | 0.047 | -0.102 |  |  |  |  |  |  |  |
| **EL** | -0.230 | -0.112 | -0.306 | **-0.564** | **-0.540** | 0.259 | 0.227 | **-0.557** |  |  |  |  |  |  |
| **MDA Leaf** | **-0.696** | **-0.628** | **-0.567** | -0.292 | -0.328 | **-0.365** | 0.010 | **-0.774** | 0.275 |  |  |  |  |  |
| **MDA Root** | **0.467** | **0.429** | 0.286 | **0.343** | 0.173 | -0.068 | 0.076 | **0.659** | **-0.551** | **-0.464** |  |  |  |  |
| **DW Leaf** | **-0.528** | **-0.528** | **-0.573** | **-0.582** | **-0.473** | -0.064 | 0.006 | **-0.914** | **0.557** | **0.717** | **-0.673** |  |  |  |
| **DW Root** | **-0.637** | **-0.551** | **-0.461** | **-0.459** | **-0.422** | -0.053 | 0.074 | **-0.920** | **0.522** | **0.713** | **-0.666** | **0.866** |  |  |
| **PRO Leaf** | -0.187 | -0.293 | 0.034 | -0.048 | -0.255 | 0.061 | 0.236 | **-0.548** | 0.249 | **0.349** | **-0.344** | **0.585** | **0.598** |  |
| **PRO Root** | **-0.511** | **-0.492** | -0.135 | -0.117 | **-0.351** | -0.066 | 0.248 | **-0.782** | **0.348** | **0.536** | **-0.485** | **0.718** | **0.742** | **0.749** |

**Supplementary Material Table 5.** Pearson correlation coefficients between morphological, biochemical parameters and chlorophyll *a* fluorescence parameters. The display option used is blue-red, which presents a negative correlation with cold colors (blue for close to -1) and positive correlations with warm colors (red for close to 1). Values in bold are different from 0 with a significance level α=0.05.

| **Variables** | **Shoot** | **Root** | **Chl*a*** | **Chl*b*** | **Car** | **Chl(*a*/*b*)** | **Chl/Car** | **RWC** | **EL** | **MD _Leaf_** | **MD _Root_** | **DW _Leaf_** | **DW _Root_** | **PR _Leaf_** | **PR _Root_** |
| --- | --- | --- | --- | --- | --- | --- | --- | --- | --- | --- | --- | --- | --- | --- | --- |
| **Fo/Fm** | -0.067 | -0.163 | -0.020 | 0.149 | 0.150 | -0.167 | -0.167 | 0.284 | -0.279 | -0.002 | **0.351** | -0.298 | -0.298 | **-0.386** | **-0.380** |
| **V_L_** | -0.217 | -0.245 | -0.176 | -0.040 | 0.003 | -0.180 | -0.132 | 0.019 | -0.066 | 0.293 | 0.118 | 0.028 | -0.034 | -0.188 | -0.159 |
| **V_K_** | 0.192 | 0.121 | 0.199 | 0.203 | 0.046 | -0.015 | 0.064 | 0.320 | -0.208 | -0.187 | **0.412** | -0.284 | -0.309 | -0.250 | -0.236 |
| **V_J_** | **0.627** | **0.562** | **0.360** | 0.183 | -0.004 | 0.205 | 0.211 | **0.527** | -0.139 | **-0.571** | **0.526** | **-0.473** | **-0.434** | -0.118 | -0.300 |
| **V_I_** | **-0.518** | **-0.473** | **-0.342** | -0.289 | -0.182 | -0.104 | -0.079 | **-0.396** | 0.076 | **0.461** | -0.240 | **0.424** | **0.457** | 0.117 | 0.315 |
| **Sm** | **-0.603** | **-0.626** | -0.181 | -0.017 | -0.131 | -0.152 | 0.054 | **-0.577** | 0.251 | **0.530** | **-0.515** | **0.457** | **0.521** | **0.396** | **0.556** |
| **N** | **-0.622** | **-0.669** | -0.130 | 0.056 | -0.124 | -0.176 | 0.077 | **-0.533** | 0.203 | **0.528** | **-0.436** | **0.410** | **0.473** | **0.344** | **0.538** |
| **Sm/t(Fm)** | -0.243 | **-0.536** | -0.294 | -0.150 | -0.136 | -0.154 | -0.093 | **-0.346** | 0.255 | **0.375** | -0.237 | **0.371** | 0.311 | 0.254 | 0.252 |
| **ABS/RC** | 0.151 | 0.070 | 0.167 | 0.205 | 0.071 | -0.046 | 0.020 | **0.335** | -0.238 | -0.162 | **0.428** | -0.307 | **-0.329** | -0.297 | -0.283 |
| **RC_i_** | -0.011 | 0.186 | 0.097 | 0.224 | 0.245 | -0.144 | -0.125 | 0.304 | -0.234 | -0.158 | 0.293 | **-0.460** | **-0.441** | **-0.663** | -0.317 |
| **DI_o_/RC** | 0.050 | -0.043 | 0.086 | 0.190 | 0.115 | -0.107 | -0.073 | **0.334** | -0.278 | -0.094 | **0.421** | -0.325 | **-0.339** | **-0.366** | **-0.356** |
| **TR_o_/RC** | 0.192 | 0.121 | 0.199 | 0.203 | 0.046 | -0.015 | 0.064 | 0.320 | -0.208 | -0.187 | **0.412** | -0.284 | -0.309 | -0.250 | -0.236 |
| **ET_o_/RC** | **-0.485** | **-0.477** | -0.209 | -0.030 | 0.042 | -0.215 | -0.167 | -0.275 | -0.026 | **0.427** | -0.204 | 0.251 | 0.190 | -0.094 | 0.104 |
| **RE_o_/RC** | **0.605** | **0.530** | **0.429** | **0.380** | 0.206 | 0.096 | 0.103 | **0.541** | -0.175 | **-0.545** | **0.423** | **-0.550** | **-0.598** | -0.234 | **-0.423** |
| **φ(P_o_)** | 0.067 | 0.163 | 0.020 | -0.149 | -0.150 | 0.167 | 0.167 | -0.284 | 0.279 | 0.002 | **-0.351** | 0.298 | 0.298 | **0.386** | **0.380** |
| **ψ(E_o_)** | **-0.627** | **-0.562** | **-0.360** | -0.183 | 0.004 | -0.205 | -0.211 | **-0.527** | 0.139 | **0.571** | **-0.526** | **0.473** | **0.434** | 0.118 | 0.300 |
| **φ(E_o_)** | **-0.600** | **-0.518** | **-0.348** | -0.207 | -0.023 | -0.169 | -0.176 | **-0.569** | 0.189 | **0.559** | **-0.581** | **0.520** | **0.481** | 0.189 | **0.365** |
| **δ(R_o_)** | **0.673** | **0.610** | **0.425** | 0.314 | 0.146 | 0.163 | 0.152 | **0.539** | -0.122 | **-0.604** | **0.413** | **-0.538** | **-0.547** | -0.148 | **-0.378** |
| **φ(R_o_)** | **0.511** | **0.477** | **0.335** | 0.265 | 0.162 | 0.119 | 0.094 | **0.356** | -0.043 | **-0.448** | 0.196 | **-0.382** | **-0.414** | -0.074 | -0.269 |

**Supplementary Material Table 6.** Pearson correlation coefficients between morphological, biochemical parameters and chlorophyll *a* fluorescence parameters. The display option used is blue-red, which presents negative correlations with cold colors (blue for close to -1) and positive correlations with warm colors (red for close to 1). Values in bold are different from 0 with significance level α=0.05.

| **Variables** | **Shoot** | **Root** | **Chl*a*** | **Chl*b*** | **Car** | **Chl(*a*/*b*)** | **Chl/Car** | **RWC** | **EL** | **MDA_Leaf_** | **MDA_Root_** | **DW_Leaf_** | **DW_Root_** | **PRO_Leaf_** | **PRO _Root_** |
| --- | --- | --- | --- | --- | --- | --- | --- | --- | --- | --- | --- | --- | --- | --- | --- |
| **ABS/CS_o_** | **0.678** | **0.675** | 0.244 | **0.373** | **0.404** | -0.139 | -0.216 | **0.712** | **-0.393** | **-0.590** | **0.626** | **-0.619** | **-0.734** | **-0.348** | **-0.548** |
| **DIo/CS_o_** | **0.499** | **0.455** | 0.175 | **0.346** | **0.371** | -0.179 | -0.236 | **0.672** | **-0.419** | **-0.457** | **0.640** | **-0.606** | **-0.696** | **-0.438** | **-0.590** |
| **TRo/CS_o_** | **0.709** | **0.719** | 0.257 | **0.367** | **0.400** | -0.121 | -0.201 | **0.698** | **-0.371** | **-0.610** | **0.597** | **-0.600** | **-0.718** | -0.306 | **-0.515** |
| **ETo/CS_o_** | 0.282 | **0.344** | -0.014 | 0.255 | **0.456** | -0.305 | **-0.409** | **0.352** | -0.307 | -0.219 | 0.236 | -0.282 | **-0.457** | -0.247 | **-0.333** |
| **REo/CS_o_** | **0.695** | **0.670** | **0.360** | **0.377** | 0.324 | 0.015 | -0.050 | **0.618** | -0.238 | **-0.615** | **0.453** | **-0.585** | **-0.668** | -0.240 | **-0.476** |
| **ABS/CS_m_** | **0.719** | **0.775** | 0.264 | 0.298 | **0.332** | -0.044 | -0.127 | **0.560** | -0.245 | **-0.597** | **0.425** | **-0.459** | **-0.574** | -0.131 | **-0.338** |
| **DIo/CS_m_** | **0.678** | **0.675** | 0.244 | **0.373** | **0.404** | -0.139 | -0.216 | **0.712** | **-0.393** | **-0.590** | **0.626** | **-0.619** | **-0.734** | **-0.348** | **-0.548** |
| **TRo/CS_m_** | **0.695** | **0.762** | 0.256 | 0.267 | 0.300 | -0.020 | -0.101 | **0.498** | -0.199 | **-0.571** | **0.359** | **-0.400** | **-0.510** | -0.075 | -0.274 |
| **ETo/CS_m_** | 0.295 | **0.412** | 0.008 | 0.147 | **0.332** | -0.171 | -0.273 | 0.151 | -0.116 | -0.200 | -0.004 | -0.079 | -0.238 | 0.011 | -0.075 |
| **REo/CS_m_** | **0.674** | **0.681** | **0.349** | 0.316 | 0.269 | 0.064 | -0.003 | **0.507** | -0.147 | **-0.584** | 0.325 | **-0.473** | **-0.552** | -0.121 | **-0.349** |
| **RC/CS** | **0.584** | **0.638** | 0.151 | 0.251 | **0.372** | -0.100 | -0.232 | **0.511** | -0.248 | **-0.502** | **0.346** | **-0.437** | **-0.539** | -0.154 | **-0.376** |
| **PI_abs_** | **-0.486** | **-0.380** | -0.298 | -0.227 | -0.069 | -0.093 | -0.097 | **-0.584** | 0.266 | **0.479** | **-0.621** | **0.535** | **0.512** | 0.311 | **0.442** |
| **PI_tot_** | 0.190 | 0.228 | 0.120 | 0.058 | 0.058 | 0.093 | 0.058 | -0.028 | 0.155 | -0.142 | -0.182 | -0.018 | -0.045 | 0.141 | 0.031 |
| **DF_abs_** | **-0.499** | **-0.397** | -0.311 | -0.238 | -0.063 | -0.094 | -0.113 | **-0.578** | 0.256 | **0.480** | **-0.621** | **0.533** | **0.510** | 0.288 | **0.422** |
| **DF_tot_** | 0.202 | 0.235 | 0.131 | 0.089 | 0.085 | 0.075 | 0.046 | -0.008 | 0.117 | -0.149 | -0.170 | -0.033 | -0.064 | 0.129 | 0.025 |
| **γ(RC)/(1-γ(RC))** | -0.145 | -0.061 | -0.160 | -0.204 | -0.075 | 0.052 | -0.012 | **-0.337** | 0.238 | 0.159 | **-0.425** | 0.305 | **0.335** | 0.312 | 0.289 |
| **φ(P_o_)/(1-φ(P_o_))** | 0.058 | 0.160 | 0.016 | -0.157 | -0.154 | 0.171 | 0.167 | -0.290 | 0.285 | 0.008 | **-0.356** | 0.302 | 0.301 | **0.382** | **0.378** |
| **ψ(E_o_)/(1-ψ(E_o_))** | **-0.634** | **-0.564** | **-0.353** | -0.173 | -0.006 | -0.211 | -0.194 | **-0.541** | 0.156 | **0.579** | **-0.534** | **0.482** | **0.445** | 0.132 | 0.328 |
| **δR_o_/(1-δR_o_)** | **0.668** | **0.608** | **0.421** | 0.312 | 0.147 | 0.162 | 0.149 | **0.542** | -0.117 | **-0.605** | **0.412** | **-0.539** | **-0.549** | -0.162 | **-0.390** |

**Supplementary Material Figure 1.** Hierarchical cluster dendrogram. A sharp increase in the value of the distance measure indicates a lower association between the clusters that merge.

**Supplementary Material Figure 3.** Hedges bias-corrected effect size (with confidence interval) of PEG-induced drought on variable fluorescence at L-band (V_L_), variable fluorescence at K-band (V_K_), variable fluorescence at J-step (V_J_), and I-step (V_I_), and the flux of energy dissipated per active PSII (DI_o_/RC) in four obtained Clusters of winter wheat genotypes (for explanation of Hedges d effect size see Sawilowsky (2009a, 2009b) and Sawilowsky et al. (2011). The ratio of total dissipation energy per active reaction center (DI_0_/RC) increases because of the high dissipation rate of active reaction centers. As the number of inactive reaction centers increases, DI_0_/RC also increases because inactive centers cannot catch a photon, resulting in an increase in the number of free photons. If a JIP test is applied, then the increase in the effective dissipation energy of the active reaction center (DI_0_/RC) also reflects the loss of connectivity between heterogeneous units of PSII, which agrees with other results (i.e. the appearance of the L-band).

References

Sawilowsky, S. (2009a). New Effect Size Rules of Thumb. *J. Mod. Appl. Stat. Methods* 8, 597–599. doi:10.22237/jmasm/1257035100.

Sawilowsky, S. (2009b). Very large and huge effect sizes. *J. Mod. Appl. Stat. Methods* 8, 597–599.

Sawilowsky, S., Sawilowsky, J., and Grissom, R. (2011). “Effect Size,” in, 426–429. doi:10.1007/978-3-642-04898-2_226.
